# Hyaluronic acid-graphene oxide quantum dots nanoconjugate as dual purpose drug delivery and therapeutic agent in meta-inflammation

**DOI:** 10.1186/s12951-023-02015-w

**Published:** 2023-08-01

**Authors:** Kunal Sarkar, Sarbashri Bank, Arindam Chatterjee, Koushik Dutta, Anwesha Das, Santanu Chakraborty, Nirvika Paul, Jit Sarkar, Sriparna De, Sudakshina Ghosh, Krishnendu Acharyya, Dipankar Chattopadhyay, Madhusudan Das

**Affiliations:** 1 Department of Zoology, University of Calcutta, 35 Ballygunge Circular Road, Kolkata, 700019 India; 2 Department of Polymer Science and Technology, University of Calcutta, 92 A.P.C. Road, Kolkata, 700009 India; 3 Department of Allied Health Sciences, Brainware University, Kolkata, 700129 India; 4 Department of Zoology, Vidyasagar College for Women, Kolkata, 700006 India; 5 Molecular and Applied Mycology and Plant Pathology Laboratory, Department of Botany, University of Calcutta, Kolkata, 700019 India

**Keywords:** Graphene oxide quantum dots, Hyaluronic acid, Metformin, Nanocomposite, Meta-inflammation, Adipose tissue inflammation, Hepatic steatosis

## Abstract

Type 2 diabetes mellitus (T2DM) predominantly considered a metabolic disease is now being considered an inflammatory disease as well due to the involvement of meta-inflammation. Obesity-induced adipose tissue inflammation (ATI) is one of the earliest phenomena in the case of meta-inflammation, leading to the advent of insulin resistance (IR) and T2DM. The key events of ATI are orchestrated by macrophages, which aggravate the inflammatory state in the tissue upon activation, ultimately leading to systemic chronic low-grade inflammation and Non-Alcoholic Steatohepatitis (NASH) through the involvement of proinflammatory cytokines. The CD44 receptor on macrophages is overexpressed in ATI, NASH, and IR. Therefore, we developed a CD44 targeted Hyaluronic Acid functionalized Graphene Oxide Quantum Dots (GOQD-HA) nanocomposite for tissue-specific delivery of metformin. Metformin-loaded GOQD-HA (GOQD-HA-Met) successfully downregulated the expression of proinflammatory cytokines and restored antioxidant status at lower doses than free metformin in both palmitic acid-induced RAW264.7 cells and diet induced obese mice. Our study revealed that the GOQD-HA nanocarrier enhanced the efficacy of Metformin primarily by acting as a therapeutic agent apart from being a drug delivery platform. The therapeutic properties of GOQD-HA stem from both HA and GOQD having anti-inflammatory and antioxidant properties respectively. This study unravels the function of GOQD-HA as a targeted drug delivery option for metformin in meta-inflammation where the nanocarrier itself acts as a therapeutic agent.

**Graphical Abstract:**

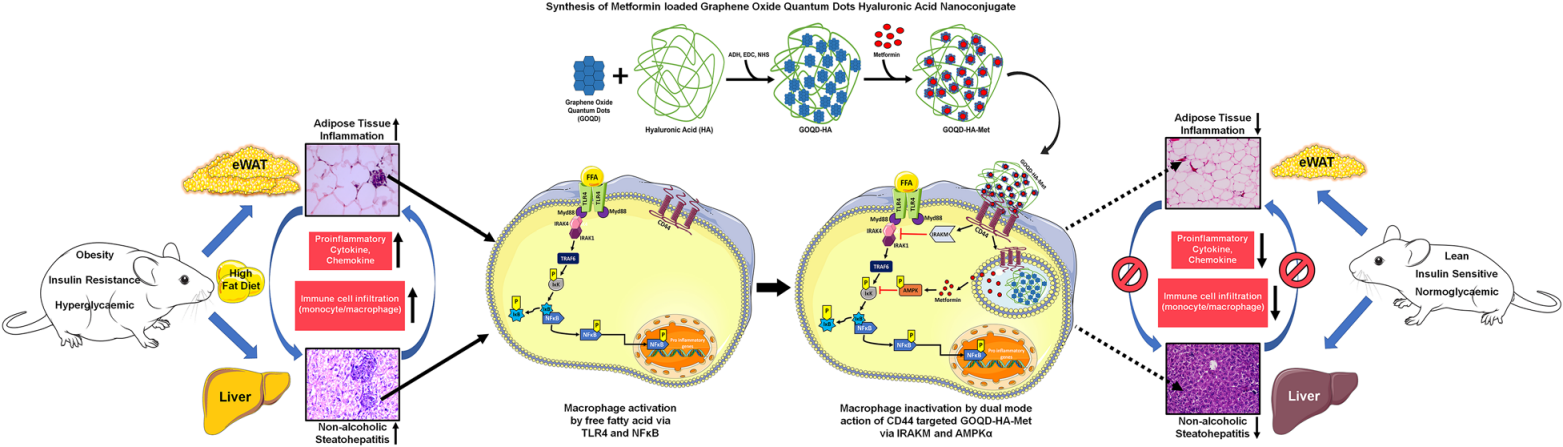

**Supplementary Information:**

The online version contains supplementary material available at 10.1186/s12951-023-02015-w.

## Introduction

Type-2 diabetes mellitus (T2DM) is one of the major contributors to death associated with the non-communicable disease worldwide. T2DM is characterized by impaired glucose homeostasis along with the inability to respond to insulin, also known as insulin resistance (IR) [1]**.** Obesity is one of the major pathophysiological risk factors involved in the advent and progression of insulin resistance and T2DM. Other metabolic, cardiovascular, and chronic inflammatory disorders such as dyslipidemia, non-alcoholic fatty liver disease (NAFLD), hypertension, coronary heart disease, and stroke are also more likely to develop in people who are obese [2, 3]. Obesity is a condition where there is an abnormal level of fat accumulation occurs in the adipose tissue (AT) due to impaired energy homeostasis and elevated energy uptake in the body [4]. As a result of continual stress induced by excess energy intake along with other factors such as immune cell infiltration, lipolysis, oxidative stress, and hypoxia, a state of chronic low-grade inflammation ensues in adipose tissue [5]. Inflammatory macrophage infiltration in adipose tissue is one of the major hallmarks of adipose tissue inflammation (ATI) in obese conditions and it is stimulated by activated adipose tissue macrophages (ATMs) that are in turn activated by adipokines produced by the adipocytes and as well as a higher level of oxidative stress [6]. These macrophages produce pro-inflammatory cytokines that ultimately result in a progression to a meta-inflammatory or chronic low-grade systemic inflammatory state [7]. Empirical evidence showed that meta-inflammation drives severe damage and promotes the threatening metabolic syndrome i.e. triad of hyperglycemia, hypertension, and dyslipidemia [8]. On the other hand, liver inflammation also progresses due to this meta-inflammation in obesity leading to non-alcoholic steatohepatitis (NASH) by the involvement of activated Kupffer’s cells and activated macrophage infiltration in the liver [9]. ATI and NASH in obese conditions are often linked with the advent of insulin resistance where the pro-inflammatory cytokines and chemokines directly or indirectly exert a negative influence on the insulin signaling pathway [10]. At present lifestyle modification/surgery are the only viable option for treating obesity and the T2DM treatment strategy is mainly focused on the improvement of metabolic parameters [11]. As chronic low-grade inflammation of AT is the common link between obesity and IR, managing the inflammation can be a potent therapeutic avenue that can target both obesity and T2DM [3], as the application of anti-inflammatory drugs has been shown to reverse insulin resistance in obese murine and human subjects. Nanoparticulate targeted therapy might be more successful in this regard as it provides an opportunity to customize the drug delivery system to actively target the sites of meta-inflammation and improve metabolic parameters as well.

Macrophages express the cell surface marker CD44 which is overexpressed in the case of inflammation thus, providing us with an opportunity to actively target sites of inflammation [12, 13]. CD44 is proven to be upregulated in T2DM, adipose inflammation, and NASH in studies involving both human and murine models [14–17]. Additionally, the blockade of CD44 with an antiCD44 antibody has been proven to reverse adipose inflammation and hepatic steatosis in mice [18]. Hyaluronic acid (HA) is an unbranched glycosaminoglycan polymer and is a ligand of CD44 [19]. HA has been used in biomedical applications owing to its outstanding characteristics such as biocompatibility, and biodegradability [20–22] and it also shows anti-inflammatory effects depending on the molecular weight of the polymer. There are several reports where high molecular weight hyaluronic acid (HMW-HA) and its nanoparticulate derivatives have been used as therapeutic agents in inflammatory diseases and wound healing [23–25]. On the other hand, graphene oxide quantum dots are 2-to-3-layer thick segments derived from graphene oxide with a size range of 1–10 nm [26]. GOQD is being extensively studied for various biological applications such as drug delivery, bio-imaging, and theranostics purposes since their discovery owing to its impeccable physical and chemical properties [27, 28]. Being carbon-based material GOQDs are chemically stable, can easily be functionalized with different chemical moieties, has low toxicity, and shows excellent biocompatibility compared to their other metallic counterparts [29, 30]. Apart from that the planar structure of GOQD offers a greater surface area for drug loading applications than any other nanoparticulate or quantum dots materials [31].

So, in this present study, we have synthesized metformin-loaded hyaluronic acid-graphene oxide quantum dot nanocomposite (GOQD-HA-Met) as a CD44 targeted therapeutic agent against meta-inflammation in obesity and T2DM. Metformin is the first line of drug for the treatment of T2DM and it also possesses anti-inflammatory effects [32–34] but it lacks targeted delivery options [35]. Conversely, metformin is placed as a BSC class-III drug due to its poor bioavailability (50–60%) thus requiring higher dosing per day (up to 2000 mg) [36]. A nanoparticulate drug delivery system is known to improve the bioavailability of such drugs due to the enhanced permeability and retention (EPR) effect [37]. There are several studies involving the application of hyaluronic acid-based graphene derivative (graphene, graphene oxide, graphene quantum dots) nanoconjugates for tumor targeting via CD44 [38–41] but study on the application of this type of conjugate in case of inflammatory diseases is an area unexplored yet. Here we propose that GOQD-HA nanocomposite is not just a mere targeted drug delivery agent but due to presence of GOQD and HA in the composition it have the potential to show antioxidant and anti-inflammatory effects respectively which can improve the efficacy of metformin for the treatment of obesity induced systemic meta-inflammation/chronic low grade inflammation and insulin resistance.

## Materials and methods

### Chemicals and reagents

Pristine graphite powder, Metformin, and Palmitic acid were purchased from Sigma. Hyaluronic acid (Abcam), adipic acid di-hydrazide (ADH) (SRL), and DCFDA (SRL) were purchased. All other chemicals and solvents used in this study were of analytical grade.

### Synthesis and characterization

#### Synthesis of graphene oxide quantum dots

First graphene oxide (GO) was synthesized from graphite by the solvothermal method. In brief 2 g of pristine graphite powder was mixed with 180 ml H_2_SO_4_ and 20 ml H_3_PO_4_ and stirred in a magnetic stirrer for ½ hr. Then 10 g of KMnO_4_ was added gradually into the mixture and the reaction continued for 4 h in the ice bath. After that, the reaction mixture was subjected to heat treatment in an oil bath at 60 °C for 6 h. Then the mixture was poured carefully into 300 ml ice-cold TDW. After that 3 ml H_2_O_2_ was added to the mixture and stirred for ½ hr. The as prepared GO was washed several times with deionized water for the elimination of unwanted salts and acid. For the preparation of GOQDs, the as synthesized GO (200 mg) was first mixed with 200 ml of HNO_3_ and sonicated for 1 h. Then the mixture was subjected to heat reflux at 95 °C in an oil bath for 8 h. After the reaction, the acid was removed by dialysis in deionized water for 3 days to obtain the GOQDs. Next, these GOQDs were lyophilized into a dry powder for further use.

#### Preparation of GOQD-Hyaluronic acid (GOQD-HA) nanocomposite

The grafting of hyaluronic acid (2000 kDa) with the GOQD was carried out via carbamide reaction in EDC and NHS reaction medium with adipic acid di-hydrazide (ADH) as a crosslinker. First, 100 mg GOQD was dispersed in 50 ml deionized for 2 h. Then 344 mg NHS and 1600 mg EDC were added to the GOQD dispersion and stirred for 3 h to activate the carboxylic groups of GOQDs. After that, 25 mg ADH was added to the mixture and stirred overnight in dark condition. The resultant GOQD-ADH is then purified by dialysis with deionized water. Next 30 mg hyaluronic acid was dissolved in citrate buffer pH 4.5, mixed with 100 mg GOQD-ADH along with NHS and EDC and stirred overnight to synthesize GOQD-HA nanocomposite. Then the resultant nanocomposite was dialyzed to remove salts and unbound HA followed by lyophilization to obtain a fine powder.

#### Preparation of metformin-loaded GOQD-HA (GOQD-HA-Met)

**Fig. 1:**
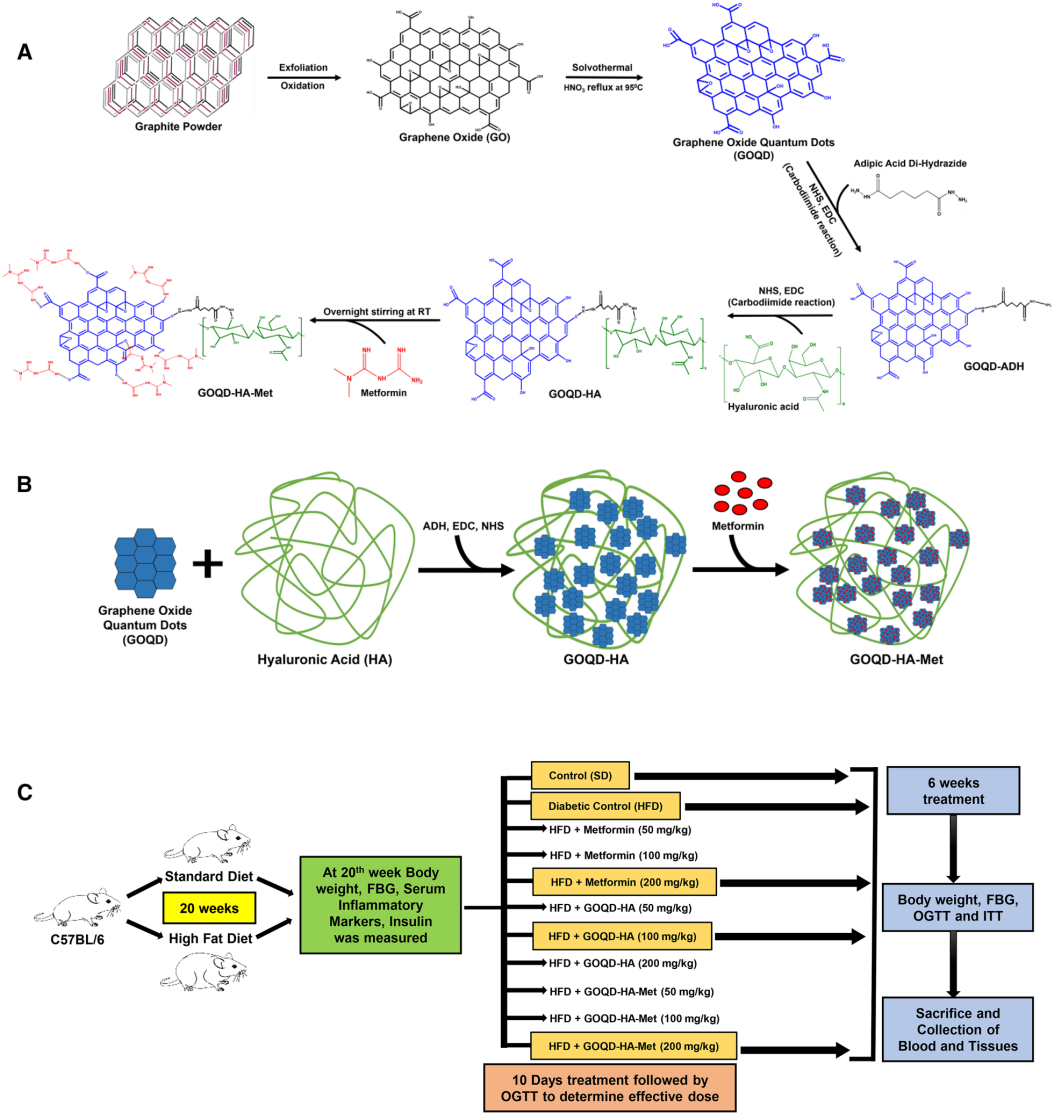
**A**. Synthesis scheme of Metformin loaded Graphene Oxide Quantum dots- Hyaluronic acid nanocomposite (GOQD-HA-Met). **B**. Simplified synthesis scheme of GOQD-HA-Met. **C**. Schematic representation of animal study design

Metformin was loaded onto the GOQD-HA by stirring GOQD-HA (50 mg) with Metformin Hydrochloride (50 mg) overnight to obtain GOQD-HA-Met. After that, the mixture was centrifuged at 10000 rpm for 10 min and the supernatant was collected for the calculation of unbound Metformin. The precipitate was collected and lyophilized for further use. The synthesis scheme for the preparation of GOQD-HA-Met starting from graphite powder is described in Fig. 1A and B.

### Characterization

The synthesized GO, GOQD, GOQD-HA, and GOQD-HA-Met were characterized by Transmission electron microscopy, Atomic Force microscopy, X-ray diffraction, Raman spectroscopy, Fourier Transformed Infra-Red Spectroscopy, UV vis spectroscopy, PL spectroscopy and Dynamic light scattering.

### Drug loading estimation

The supernatant collected after the centrifugation step mentioned in Sect. “Preparation of Metformin-loaded GOQD-HA (GOQD-HA-Met)” was subjected to UV–Vis spectrophotometry at 234 nm for the determination of unbound metformin concentration. The drug loading was calculated using the formula:

$$Drug~Loading~\% = \frac{{Total~amount~of~drug - amount~of~drug~in~supernatant}}{{Total~amount~of~drug}}$$

### Drug release profile

The drug release profile of metformin from GOQD-HA-Met was evaluated by dialysis method at 3 physiological pH conditions (pH 2, 5.6, and 7.4) and pH 12. In brief 50 mg of GOQD-HA-Met was dispersed in 25 ml of TDW and loaded inside the dialysis membrane (mol. wt. cutoff 2000 kDa) and the membrane was submerged in the buffer having the aforementioned pH levels. At each hour for 24 h, 2 ml of the buffer solution was pipetted out and the same amount was replenished. The pipetted-out buffer solution was then analyzed by UV–Vis spectrophotometer for the detection of released metformin at 234 nm.

### Cell culture

RAW 264.7 cell line was acquired from NCCS, Pune, India, and the cells were cultured in DMEM media with 10% FBS, and 1% penicillin–streptomycin at 37 °C and 5% CO_2_.

#### Cytotoxicity evaluation of GOQD-HA and GOQD-HA-Met

MTT assay was performed to evaluate the cytotoxicity of GQD-HA and GQD-HA-Met. In brief, RAW264.7 cells were seeded in 96 well plates with a 5 × 10^4^ number and incubated in different concentrations (50 µg/ml to 800 µg/ml) of GQD-HA and GQD-HA-Met for 24 h. Afterward, cells were washed three times with PBS and MTT dye (5 mg/ml) was added to each well and the plate was incubated at 37 °C for 4 h. After that, supernatants were discarded, 200 µl DMSO was added to each well and was shaken lightly for 10 min to dissolve formazan crystals. The absorbance was recorded at 570 nm using Varioskan LUX Multimode Microplate Reader (Thermo Fisher Scientific).

We further investigated the biocompatibility of GOQD-HA and GOQD-HA-Met by live/dead staining assay by fluorescence microscopy [42, 43] with some modifications. Here, cells were seeded in 35 mm tissue culture dishes and treated for 24 h according to the dose variation described in the MTT assay section. After that cells were washed thrice with PBS and fresh serum free media containing fluorescein diacetate (FDA) and propidium iodide was added and incubated for 30 min. Then cells were washed 5 times with PBS and observed under fluorescence microscope for the evaluation of cytotoxicity of the nanoconjugates.

#### Intracellular uptake of GOQD-HA-Met

RAW 264.7 cells were divided into untreated control and palmitic acid (250 µM) treated groups. Both groups were incubated with 65 µg/ml GOQD-HA-Met for 8 h, washed thrice with ice-cold PBS, and then fixed with chilled methanol and cells were subjected to immunofluorescence against CD44 following protocol detailed in Sect. 2.5.6. The cells were then observed under a confocal laser scanning microscope (Olympus).

#### In vitro optimum dose selection

Firstly, for dose selection cells were seeded on 96 well plates and treated according to the following 11 groupings: Normal Control, Palmitic acid (250 µM), PA + Metformin (0.25 mM), PA + Metformin (0.5 mM), PA + Metformin (1 mM), PA + GOQD-HA (32.5 µg/ml), PA + GOQD-HA (65 µg/ml), PA + GOQD-HA (High) (130 µg/ml), PA + GOQD-HA-Met (65 µg/ml; eqv. 0.25 mM Metformin), PA + GOQD-HA-Met (130 µg/ml; eqv. 0.5 mM Metformin), PA + GOQD-HA-Met (260 µg/ml; eqv. 1 mM Metformin) for 8 h. Then ROS generation was evaluated by DCFDA assay to determine the optimum dosing of Metformin, GOQD-HA and GOQD-HA-Met for further elaborate in vitro experiments. Afterward, cells were treated according to the following groups: Normal Control (Control), Palmitic Acid 250 μM (PA), PA 250 μM + Metformin 1 mM (PA + Met), PA 250 μM + GOQD-HA 130 µg/ml (PA + GH), PA 250 μM + GOQD-HA-Met 130 µg/ml (PA + GHM) for further experiments.

#### Intracellular ROS determination by DCFDA assay via fluorescence microscopy

Intracellular ROS generation was measured by DCFDA staining according to [44]. Cells were divided into five groups and treated according to the doses as previously mentioned in Sect. “In vitro optimum dose selection” for 8 h. After treatment cells were again incubated with dichloro-dihydro-fluorescein diacetate (DCHF-DA) for 30 min in the dark condition at 37 °C. The cells were then washed with PBS thrice and observed under a fluorescence microscope.

#### Immunofluorescence

RAW 264.7 cells were cultured on poly-L-Lysine coated coverslips for 24 h. Next, after the following treatment as specified in Sect. “In vitro optimum dose selection”, cells were washed three times with PBS and then fixed with 4% paraformaldehyde for 10 min. Again, cells were washed with ice-cold PBS three times, and cells were blocked in 1% BSA for 2 h. Then, cells were washed with PBS 5 times and incubated overnight with primary antibody anti-CD44 and anti-phospho p65 antibody, followed by incubation with anti-rabbit Alexa Fluor 488 tagged secondary antibody for 2 h. Further, cells were washed with PBS 5 times, stained with DAPI, and examined under a fluorescence microscope (Olympus).

### Animals

C57BL/6 mice were provided by the West Bengal Live Stock Development Corporation, West Bengal, India. Mice were maintained with food and water ad libitum and were kept in polypropylene cages with consistent 12-h light/dark cycle at constant temperature (22–25 °C) with a relative humidity of 60–70% in the animal house room for acclimatization. All the animal studies were performed as per the approved protocol of the institutional animal ethics committee of the Department of Zoology, University of Calcutta.

#### In vivo optimum dose selection and experimental design

The diabetic model was established by feeding mice with a high fat diet (HFD) for 20 weeks while the control group was fed a standard diet (SD). At first, the mice were divided into 11 groups: Normal Control-SD fed (NC), Diabetic Control- HFD fed (DC), HFD + Metformin (50 mg/kg), HFD + Metformin (100 mg/kg), HFD + Metformin (200 mg/kg), HFD + GOQD-HA (50 mg/kg), HFD + GOQD-HA (100 mg/kg), HFD + GOQD-HA (200 mg/kg), HFD + GOQD-HA-Met (50 mg/kg; eqv. 25 mg/kg Metformin), HFD + GOQD-HA-Met (100 mg/kg; eqv. 50 mg/kg Metformin), HFD + GOQD-HA-Met (200 mg/kg; eqv. 100 mg/kg Metformin). After 10 days of oral dosing according to the abovementioned protocol mice were subjected to an oral glucose tolerance test (OGTT) for determination of optimum dosing of metformin, GOQD-HA, and GOQD-HA-Met in in vivo conditions. Next, for further experiments the mice were divided into 5 groups: Normal Control-SD fed (NC), Diabetic Control- HFD fed (DC), HFD + Metformin 200 mg/kg (HFD + Met), HFD + GOQD-HA 100 mg/kg (HFD + GH), HFD + GOQD-HA-Met 200 mg/kg; eqv. 100 mg/kg Metformin (HFD + GHM) following a chronic oral drug administration for 6 weeks (Fig. 1C). Subsequently effect of chronic treatment on hyperglycemia was determined by OGTT and ITT. Then mice were sacrificed, thereby blood plasma and tissue samples (eWAT, liver and muscle) were collected for various biochemical assays, histopathology, and expression studies.

#### Oral glucose tolerance test (OGTT) and insulin tolerance test (ITT)

OGTT was performed in 6 h fasted mice. 2 g/kg body weight glucose was challenged by oral gavage 30 min after treatment with drugs; NC and DC groups received 0.9% normal saline as treatment. Blood samples were collected from the tail vein at 0, 30, 60, 90, 120, and 180 min of treatment. Insulin Tolerance Test (ITT) was conducted by administering insulin (0.6 U/kg) intraperitoneally after a 4-h fasting. Blood samples were collected from the tail vein at 0, 30, 60, 90, and 120 min of treatment. Blood glucose levels were estimated by Accucheck Active (Roche, Germany) in both OGTT and ITT.

#### Plasma lipid profile determination

Plasma triglycerides (TG) and cholesterol (TC) were measured according to the manufacturer’s protocol provided by the commercial kit (Arkray Technologies, India).

#### Liver function test parameters

Collected serum from mice was subjected to analysis of liver function markers serum glutamic pyruvic transaminase (SGPT), serum glutamic oxaloacetic transaminase (SGOT), and alkaline phosphatase (ALP) using commercial assay kits.

#### Estimation of serum inflammatory markers and plasma insulin level

To quantify the amount of CD44 from plasma samples of mice, we conducted an indirect ELISA method as described below, samples were incubated with PBS (phosphate buffer) in 96 well ELISA plates overnight at 4 °C. Then the plates were washed with PBS (containing Tween-20) and 5 mg/ml BSA (bovine serine albumin) was used to block non-specific binding. Again, after washing the plate, primary antibody CD44 was incubated for 2 h. Next, plates were washed further with PBS-T20 buffer thrice and incubated with goat anti-mouse IgG (HRP/Alkaline phosphatase-conjugated) (1:10000) for 1 h. Next, plates were washed with PBS-T20 and incubated with the substrate OPD (1 mg/ml) in 0.05 M Citrate–Phosphate buffer. The development of color was measured at 450 nm. The levels of IL-1β, TNF-α, and IL-6 and insulin levels in the mice plasma samples were measured according to the manufacturer's protocol available with the assay kit.

#### Measurement of enzymatic and nonenzymatic antioxidants

##### Preparation of samples for enzyme assay and immunoblot analysis

The tissue was isolated and homogenized in 50 mM cold potassium phosphate buffer (PBS) (pH 7.4) containing 0.1% Triton X-100 followed by centrifugation at 15,000 g for 20 min at 4 °C. The resulting supernatants were collected and utilized for subsequent biochemical analysis and the total protein contents of the tissue were determined at a wavelength of 595 nm according to the Bradford method [45] using BSA as a protein standard.

##### Lipid peroxidation (MDA measurement)

Tissue (500–700 mg) from eWAT and liver was homogenized in 0.375% TBA (T5500 Sigma Aldrich) and 15% TCA (1:4) in 1 ml total solution, then the mixture was incubated for 40–45 min in boiling water bath. After cooling, it was centrifuged at 5000 g for 15 min and absorbance was measured at 532 nm, expressed as nanomole concentration of MDA content/mg protein. Lipid peroxidation was determined by a TBARS (thiobarbituric acid-reactive substances) assay, performed by a reaction of malondialdehyde (MDA) with 2-thiobarbituric acid (TBA), according to [46].

##### Catalase (CAT) activity

Catalase (CAT) activity of treated and control tissue was determined according to [47] by measuring the rate of H_2_O_2_ decomposition at 240 nm for 2 min at a regular 30 s interval. The reaction mixture contained 50 mM potassium phosphate buffer (pH 7) and 3% H_2_O_2_ and 50 µL supernatant. CAT activity (U/mg protein) was calculated by using the extinction coefficient of H_2_O_2_ (34.9 M^−1^ cm^−1^).

##### Superoxide dismutase (SOD) activity

The reaction mixture composed of 80 mM Tris (pH 8.9) 0.12 mM EDTA, 10.8 mM TEMED, 0.003% BSA, 30 μM riboflavin in 5 mM KOH, 300µmM NBT (SRL, 11207), 50 µL supernatant was added. It was kept under light (150W) for 2 min and the absorbance was measured at 560 nm according to [48]. A blank without enzyme and light showed the amount of NBT present in the reaction mixture. SOD was calculated as U/mg protein.

##### Measurement of total glutathione (GSH: GSSG)

Reduced glutathione (GSH) and total glutathione content were determined by [49] method. For glutathione determination, 20 µL DTNB (0.4%) was mixed with 20 µL enzyme extract containing 5-Sulphosalicylic acid in 150 µL Na-phosphate buffer (0.1 M), pH 7, resulting in the formation of GSSG and TNB (5-thionitrobenzoic acid), which was detected at 412 nm. A standard curve of GSH was generated by using 400 µM to perform serial dilution up to 50 µM and was expressed as µmole of GSH/mg protein. For estimation of total glutathione (GSH + GSSG) level, 12.5 µL NADPH and 2.5U Glutathione Reductase (GR, Merck G3664) were mixed with the previous reaction mixture containing DTNB. Then it was kept for 30 min at 37 °C, and absorbance was measured at 412 nm. The GSSG content was calculated as the difference between the total glutathione and GSH contents and then the GSH/GSSG ratio was evaluated.

### Gene expression

#### Extraction of total RNA & quantitative real-time PCR (RT-PCR) analysis

Total RNA was isolated from cell lysates and tissues using Trizol reagent (Ambion, life technologies) according to the manufacturer's instructions. Reverse transcription of 1 μg of total RNA from each sample was performed using an iScript cDNA synthesis kit. Transcript levels of inflammatory and metabolic genes from both RAW264.7 cell lysate and tissue were analyzed by quantitative real-time PCR (Applied Biosystems QuantStudio™ 5 Real-Time PCR System). The details of the forward and reverse primers for the selected genes are provided in Additional file 1: Table S1. The relative mRNA expression of the tested gene was quantified by applying the comparative 2^−ΔΔCt^ method according to Chatterjee et al. [50] using GAPDH as the reference gene.

#### Immunoblotting

Relative expression of the protein in treated animal tissue and cell lysate was detected by Western blotting. Equal amount of protein samples was prepared in 2xLaemmli buffer denatured at 95 °C for 3 min, separated by sodium dodecyl sulfate–polyacrylamide gel electrophoresis (SDS-PAGE) on a 10% gel and transferred to a PVDF membrane (0.2 µm, Bio-Rad) by Trans-Blot^®^ Turbo Transfer System (Bio-Rad). After the membrane was incubated with primary antibodies (anti-AMPK, anti-phospho-AMPK, anti-CD44, anti-IRAKM, anti-GLUT-4, anti-p65, and anti-phospho-p65, anti-α-Tubulin) and the protein bands were detected by chemiluminescence method. Densitometric quantification of Western blots was performed by utilizing ImageJ software (NIH) according.

### Histopathological studies

Liver and adipose tissues were collected from different animal groups and stored in neutral buffer formalin for 24 h, immediately after sacrifice. After the standard procedure, paraffin-embedded tissue was cut into sections (4 µm) followed by H&E staining, and then observed under a light microscope.

### Statistical analysis

All the data were analyzed by GraphPad Prism software version 9.3.1 (San Diego, CA, USA), and the results were presented as Mean ± SEM. One-way ANOVA followed by Tukey's post hoc test was used to determine statistical significance where *p < 0.05, *p < 0.01, and ***p < 0.001.

## Results

### Synthesis and characterization of metformin-loaded graphene oxide quantum dots-hyaluronic acid nanoconjugate

#### Synthesis and characterization of Graphene Oxide (GO) and Graphene Oxide Quantum Dots (GOQDs)

**Fig. 2:**
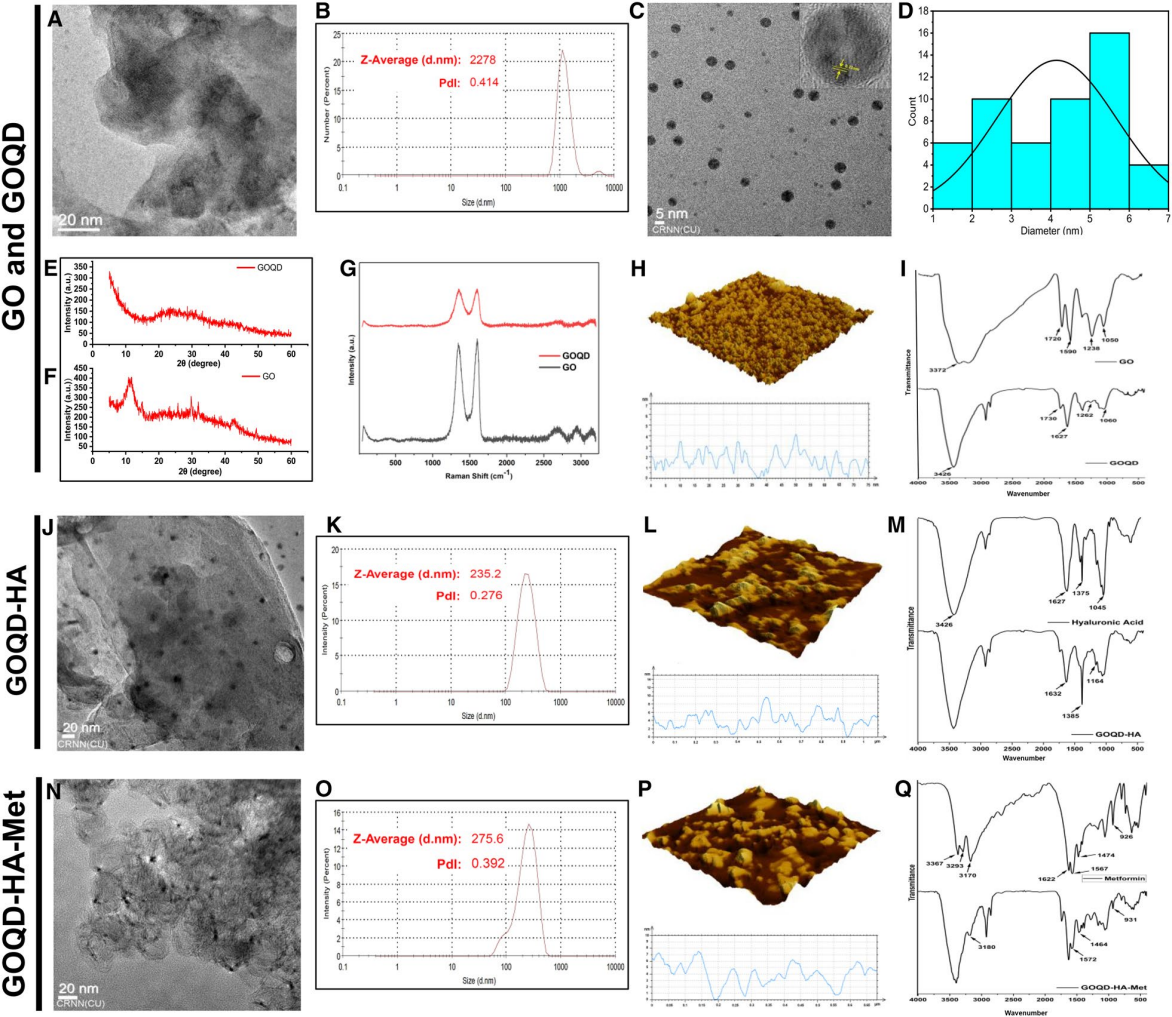
**Characterization of synthesized GO, GOQD, GOQD-HA and GOQD-HA-Met.** **A**. TEM image of GO, **B**. Particle size distribution of GO, **C**. TEM image of GOQD, **D**. Particle size distribution of GOQD, **E. and F**. X-ray diffraction pattern of GOQD and GO, **G**. Raman spectra of synthesized GOQD and GO, **H**. AFM and surface roughness of GOQD, **I**. FTIR spectra of GO and GOQD, **J**. TEM image of GOQD-HA, **K**. Particle size distribution of GOQD-HA, **L**. AFM and surface roughness of GOQD-HA, **M**. FTIR spectra of Hyaluronic acid and GOQD-HA, **N**. TEM image of GOQ-HA-Met, **O**. Particle size distribution of GOQD-HA-Met, **P**. AFM image of GOQD-HA-Met, **Q** FTIR spectra of Metformin and GOQD-HA-Met

Graphene oxide was synthesized using the solvothermal method using pristine graphite powder as starting material. The main phenomenon of GO synthesis is the introduction of oxygen containing groups and the transformation of sp^2^ bonds into sp^3^ bonds. In the structure of GO the epoxy and hydroxide groups lay on the basal surface while the carboxyl and carbonyl moieties lie on the edges of the sheets. GOQDs were prepared from GO by solvothermal method by HNO_3_ reflux at 95 °C. in GOQDs a hydroxyl, epoxy, and carboxyl-bound sp^3^ matrix wraps around the aromatic sp^2^ domains [51]. Transmission electron microscopy images (Fig. 2A) showed the GO has a thin sheet-like appearance with folds on the surface. The average hydrodynamic size of GOQD was 2278 nm as elucidated by DLS (Fig. 2B). Whereas GOQDs showed a distinct round-shaped morphology with sizes ranging from 1.3 nm to 6.5 nm (Fig. 2C). The average size distribution of GOQDs was 4.34 nm which was calculated from TEM images according to [52] (Fig. 2D). High resolution TEM images of the GOQD revealed crystalline nature with inter layer spacing of 0.11 nm. Atomic force microscopy was applied to discern the surface roughness of the synthesized GOQDs (Fig. 2H), where it was found that the GOQDs have significant crystalline morphology with the mean height of the GOQDs being 3 nm signifying the presence of 2- 3 layers of graphene in the GOQDS. A distinct X-ray diffraction peak was observed at 2θ = 11.68° for GO (Fig. 2F), while the diffraction intensity was flattened in case of GOQD with a broad and less intensity peak at 2θ = 26.23° (Fig. 2E). Raman spectra of GO revealed two distinct peaks at 1345 cm^−1^ and 1602 cm^−1^, whereas GOQD showed peaks at 1355 cm^−1^ and 1595 cm^−1^ (Fig. 2G). The peaks at 1345 cm^−1^ and 1355 cm^−1^ were assigned as D bands while 1602 cm^−1^ and 1595 cm^−1^ peaks were categorized as G bands for GO and GOQD respectively. The ID/IG ratio of GOQD (1.007) was found to be higher than GO (0.94). Also, the peaks of GOQD were broader than that of GO finding similar to previous reports [53]. FTIR spectra (Fig. 2I) revealed several characteristic peaks of GO at 3396 cm^−1^ for -OH, 1720 cm^−1^ for C = O, 1622 cm^−1^ for C = C, and 1085 cm^−1^ for C-O [41]. Similarly, in GOQD the presence of functional groups like  −  OH, C = O, C = C, and C-O were confirmed by peaks at 3416 cm^−1^,1725 cm^−1^, 1622 cm^−1^, and 1070 cm^−1^ respectively (Fig. 2I). The intensity of the peaks at 1725 cm^−1^ and 1070 cm^−1^ were significantly increased in GOQD mainly due to the addition of carboxy and carbonyl groups after solvothermal treatment of GO thus indicating the presence of more oxygen containing groups in GOQD than GO. The characteristic peak at 227 nm was observed in GO by UV–vis spectroscopy (Additional file 1**: **Fig. S1A) owing to the π-π* transition of aromatic sp^2^ domains and another low intensity shoulder at 280 nm for n-π* transition was also found. On the other hand, in GOQD the peaks for π-π* and n-π* transition was found at 227 nm and 280 nm respectively (Additional file 1: Fig. S1B). Due to the presence of a greater number of functional groups in GOQD, here the shoulder peak of 280 nm was much higher in intensity than GO as reported in previous studies [54]. The GOQDs showed strong blue fluorescence under 265 nm UV light, so we evaluated the photoluminescence spectra of GOQD (Additional file 1: Fig. S1C) with excitation wavelength ranging between 280 and 360 nm. The peak fluorescence intensity was observed for λ_ex_ 330 nm at λ_em_ 450 nm. The GOQDs were well dispersed in a water medium and stable for several months. The surface charge of the GOQDs was recorded at − 19.2 mV as evaluated by measuring zeta potential in the aqueous medium (Additional file 1: Fig. S1D). The negative zeta potential of the GOQDs is mainly due to the presence of negatively charged carboxyl, carbonyl, and hydroxyl groups.

#### Synthesis and characterization of GOQD-Hyaluronic Acid nanoconjugate (GOQD-HA)

GOQDs was functionalized by Hyaluronic acid using ADH as a crosslinker through carbodiimide chemistry. The formation of the nanoconjugate was evaluated by various methods. First, the TEM images of the synthesized GOQD-HA nanocomposite (Fig. 2J) show the presence of GOQDs embedded in the polymer. The average hydrodynamic particle size of the GOQD-HA was recorded at 235.2 nm (Fig. 2K). The morphology of the nanoconjugate was amorphous and had a mean diameter of 100–200 nm. The surface roughness of the nanoconjugate was decreased as revealed by the atomic force microscopy and the amorphous nature of the GOQD-HA was also confirmed here (Fig. 2L). Although the height of the nanoconjugate was elevated from 3–4 nm of GOQDs to 6–7 nm for the GOQD-ha mainly due to tagging of HA with the GOQD. Conjugation of hyaluronic acid with GOQDs was further confirmed by FTIR spectroscopy (Fig. 2M). After the crosslinking of HA with GOQD by carbodiimide reaction, the peak for C = O at 1725 cm^−1^ almost disappeared and the intensity of the peak for the amide-I bond at 1627 cm^−1^ increased thus signifying successful conjugation. Also, another characteristic peak at 1385 cm^−1^ for C-H bending appeared in the IR spectrum of the GOQD-HA nanoconjugate along with the skeletal acetal valence bond peak at 1164 cm^−1^. The zeta potential of the nanocomposite (GOQD-HA) moved further towards the negative side at -31.8 mV (Additional file 1: Fig. S1E) compared to GOQD due to the addition of the anionic polymer hyaluronic acid.

#### Synthesis and characterization of Metformin loaded GOQD-HA nanocomposite (GOQD-HA-Met)

Metformin was loaded onto the GOQD-HA nanoconjugate by overnight stirring at room temperature and then acetone was added to remove the hydrogen bonding of hyaluronic acid with the water. The treatment of the drug-loaded GOQD-HA with acetone led to changes in the morphology of the nanoconjugate into a globular form from amorphous nature as revealed by the TEM image (Fig. 2N). DLS study revealed the GOQD-HA-Met have an average hydrodynamic size of 275.6 nm (Fig. 2O). AFM study also revealed the globular morphology (Fig. 2P) and also an increase in the surface roughness of the GOQD-HA-Met due to the loading of metformin onto the nanoconjugate. The height of the nanoconjugate increased to 7–8 nm as was also evident in the AFM images (Fig. 2P). Metformin being a biguanide shows several characteristic peaks like 3367 cm^−1^ (N–H asymmetric stretching), 3293 cm^−1^ and 3170 cm^−1^ (N–H symmetric stretching), 1622 cm^−1^ (C = N stretching), 1567 cm^−1^ (N–H in-plane deformation), 1474 cm^−1^ and 1410 cm^−1^ (CH_2_ asymmetric deformation), 926 cm-1 (N–H wagging) [55]. IR spectra of the metformin-loaded GOQD-HA (GOQD-HA-Met) (Fig. 2Q) revealed the appearance of these abovementioned peaks (3180 cm^−1^, 1572 cm^−1^, 1464 cm^−1^, 1420 cm^−1^, 931 cm^−1^) thus confirming the presence of metformin in the formulation. After loading of metformin onto the GOQD in the GOQD-HA nanocomposite the zeta potential was brought up to -13.7 mV (Additional file 1: Fig. S1F). Metformin is a cationic drug, thus loading of metformin leads to a rise in the zeta potential to the + ve side, indicating efficient loading of the drug onto the surface of GOQD. The drug loading percentage of metformin in GOQD-HA nanoconjugate was 97.65%. The efficient drug loading was due to the formation of hydrogen bonding between metformin and GOQD and HA as metformin has 5 hydrogen bond acceptors and 3 H- bond donors present in its structure. Drug release was evaluated by dialysis method at three physiological pH conditions (pH 2, 6, and 7.4) and also at pH 12 (Additional file 1: Fig. S2). There was a burst release of 50.08% and 44% of the drug after 6 h at pH 7.4 and pH 5.6 respectively and then the release pattern forms a plateau showing a sustained release pattern. Interestingly at pH 12, the release of the drug from the nanoconjugate is 80.46% after 8 h.

### GOQD-HA-Met shows satisfactory in vitro biocompatibility and efficient uptake in RAW 264.7 cells

**Fig. 3:**
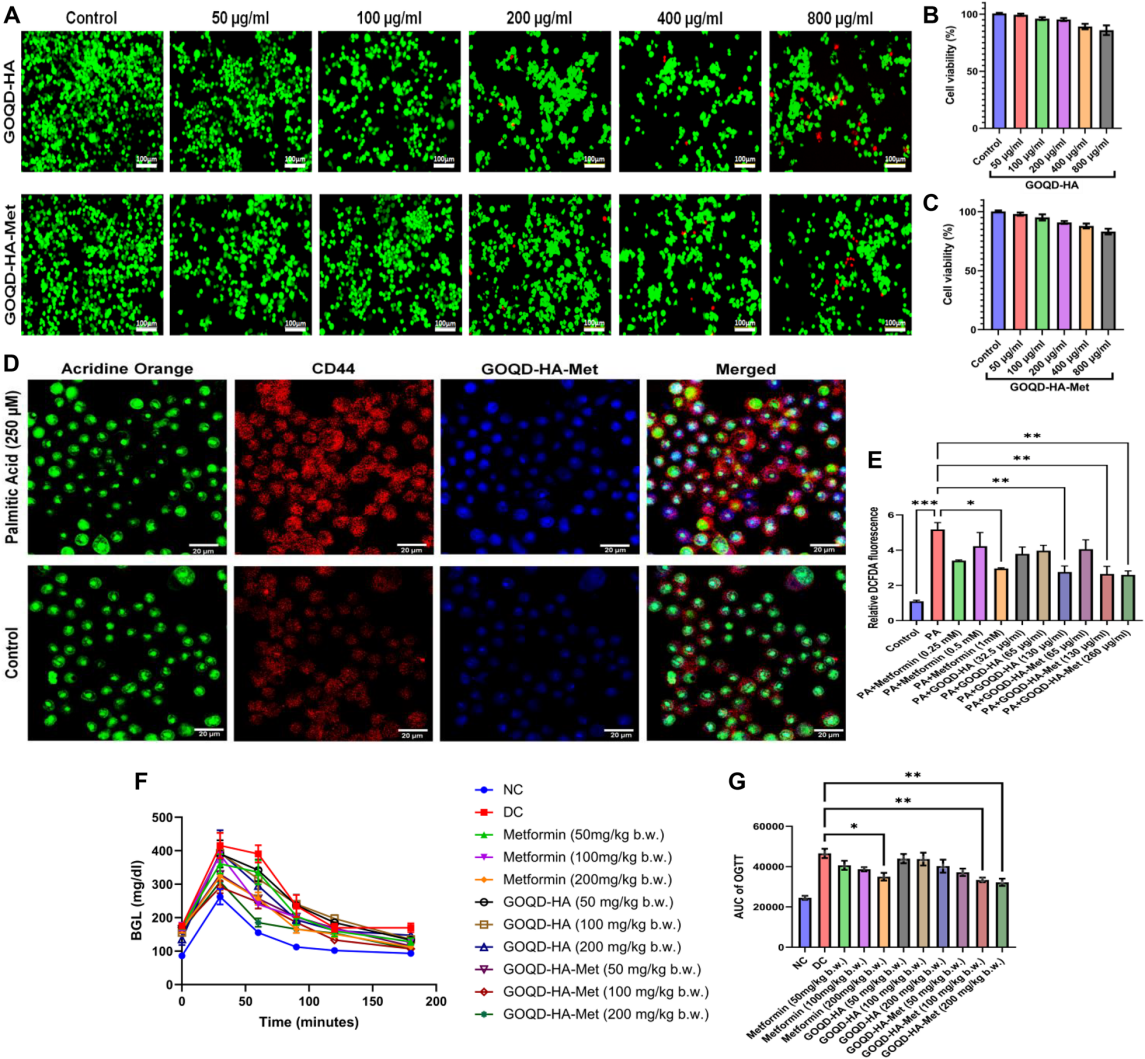
**Cytocompatibility, cellular uptake and dose optimization for GOQD-HA-Met.** **A.** Live/ Dead assay by FDA/PI staining of RAW264.7 cells treated with GOQD-HA and GOQD-HA-Met showing satisfactory cytocompatibility, **B.** and **C**. in vitro cytotoxicity evaluation by MTT assay of GOQD-HA and GOQD-HA-Met in RAW 264.7 cells. **D**. CLSM images of RAW 264.7 cells treated with GOQD-HA-Met showing enhanced uptake in PA induced cells compared to untreated group. **E**. In vitro dose optimization for metformin, GOQD-HA and GOQD-HA-Met by DCFDA assay in PA induced RAW264.7 cells showing significant decrease in DCFDA fluorescence in Metformin (1 mM), GOQD-HA (130 μg/ml) and GOQD-HA-Met (130 μg/ml and 260 μg/ml) groups. **F**. OGTT and **G**. AUC of OGTT showing in vivo dose optimization in HFD fed mice after 10 days of treatment. Metformin (200 mg/kg b.w.) and GOQD-HA-Met (100 mg/kg b.w. and 200 mg/kg b.w.) showed statistically significant improvement of oral glucose tolerance compared to diabetic control (DC) group. (Data represented as Mean ± SEM (n = 3) where *p < 0.05, **p < 0.01 and ***p < *0.001*.)

After successful synthesis of the metformin-loaded graphene oxide quantum dots – hyaluronic acid nanoconjugate the in vitro cytotoxicity was evaluated in RAW264.7 cells in a concentration range between 50 µg/ml and 800 µg/ml for both GOQD-HA (Fig. 3B) and GOQD-HA-Met (Fig. 3C) by MTT assay. Both the blank and drug-loaded nanoconjugate showed minimal toxicity with cell viability of 85% and 83% respectively at the highest dose (800 µg/ml) administered. The cell viability was further evaluated by FDA/PI staining of RAW264.7 cells (Fig. 3A) incubated with GOQD-HA and GOQD-HA-Met in the concentration range mentioned above, where similar to the MTT assay it was observed that both the drug loaded and blank nanocomposite was sufficiently biocompatible as evident by minimal number of PI stained cells even at the higher doses.

Next, we evaluated cellular uptake of the synthesized drug-loaded nanocomposite (GOQD-HA-Met) in palmitic acid-treated RAW 264.7 cells compared to untreated cells and correlated it with the expression of CD44 by immunofluorescence (Fig. 3D). The PA-induced RAW 264.7 cells showed significantly higher expression of CD44 as opposed to the untreated control cells. Interestingly, there is a significantly higher uptake of the GOQD-HA-Met in the PA treated cells as well, corresponding to the higher expression of the CD44 as compared to the control cells. Thus, this signifies the efficiency of the synthesized nanoconjugate to target and deliver the drug metformin in CD44 targeted manner.

### In vitro and in vivo dose optimization studies revealed GOQD-HA-Met enhances the efficiency of Metformin in lower doses than free Metformin

First, we optimized the dosing of the nanoconjugate for in vitro studies according to the dosing protocol discussed in Sect. “In vitro optimum dose selection”. We evaluated ROS generation by DCFDA assay in palmitic acid-treated RAW 264.7 cells with or without Metformin, GOQD-HA, and GOQD-HA-Met treatment as a marker for the efficacy of these drugs. We observed that the 65 µ g/ml GOQD-HA-Met (eqv. to 0.25 mM metformin) was able to mitigate the ROS generation upon palmitate-induced stress more efficiently in the RAW264.7 cells (Fig. 3E). On the other hand, Metformin and GOQD-HA were able to reduce the generation of ROS more efficiently at 1 mM and 130ug/ml respectively. These dosages were chosen for further elaborate experiments and divided into five following groups: Control, Palmitic acid (PA), PA + Met (Metformin, 1 nM), PA + GH (GOQD-HA, 130 µg/ml), and PA + GHM (GOQD-HA-Met, 65 µg/ml).

For in vivo studies, C57/B6 mice were fed a high fat diet for 20 weeks to develop diet induce obese mice with T2DM and IR., while mice fed with a standard chow diet were taken as the control group. After 20 weeks, fasting blood glucose (FBG) and body weight were elevated in the HFD group. We also checked for inflammatory markers IL-1b and IL-6 in the plasma of both HFD and SD fed mice. There was a significant increase in these proinflammatory cytokine levels in the HFD group.

Next, we divided the mice into 11 groups as described in  Sect. “In vivo optimum dose selection and experimental design”. and treated with metformin, GOQD-HA, and GOQD-HA-Met for 10 days followed by an oral glucose tolerance test to determine the optimal dosing for each treatment (Fig. 3F). It was observed from the AUC of OGTT (Fig. 3G) that 200 mg/kg metformin and 200 mg/kg GOQD-HA-Met (eqv. to 100 mg/kg metformin) were able to induce significant improvement in oral glucose tolerance when compared to DC group after 10 days of treatment. Interestingly the GOQD-HA dosages showed no statistically significant changes in glucose tolerance after 10 days of treatment. So, we have chosen the dosing for GOQD-HA at 100 mg/kg as this was the equivalent amount present in the 200 mg/kg GOQD-HA-Met nano-formulation. So, for further experiments the mice were divided into 5 groups with chosen doses as follows: NC (SD fed), DC (HFD fed), HFD + Met (Metformin, 200 mg/kg b.w.), HFD + GH (GOQD-HA, 100 mg/kg), HFD + GHM (GOQD-HA-Met, 200 mg/kg).

### GOQD-HA-Met ameliorates FFA-induced oxidative stress and inflammation in RAW264.7 cells

**Fig. 4:**
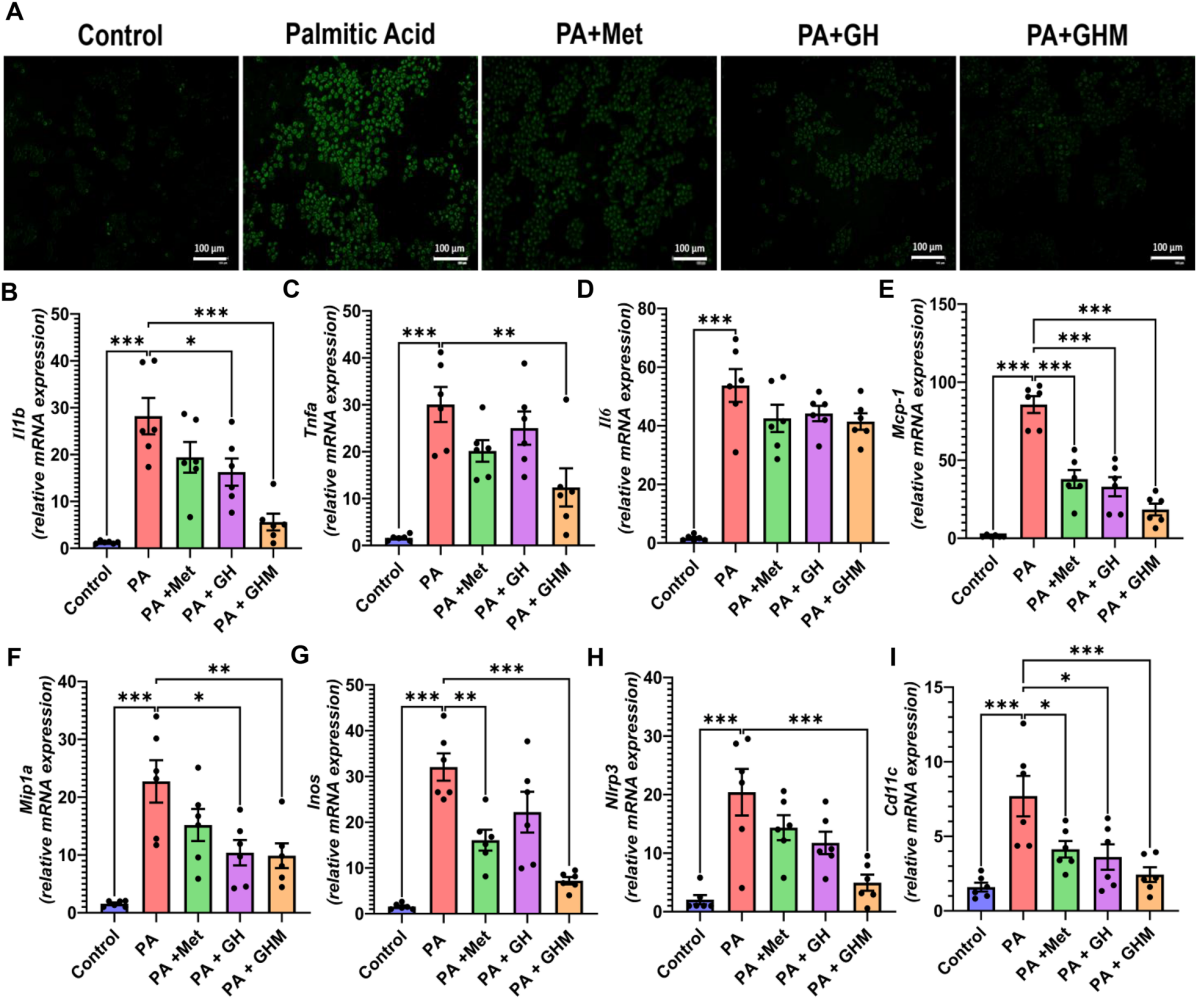
**In vitro anti-inflammatory effects of GOQD-HA-Met.** **A**. RAW264.7 cells treated with PA for 6 h showing high level of ROS generation upon DCFDA staining compared to untreated cells. GOQD-HA-Met treatment mitigated the ROS generation more significantly than free metformin and blank GOQD-HA as indicated by lowered DCFDA fluorescence. **B.**-**E**. Quantitative real time PCR showed treatment of RAW264.7 cells with PA increased the mRNA expression of proinflammatory cytokines, **B**. Il1b, **C**. Tnfa, **D**. Il6; chemokines, **E.** Mcp1, **F.** Mip1a; mediators of inflammation, **G**. Inos, **H**. Nlrp3; and proinflammatory macrophage cell surface marker, **I** Cd11c compared to untreated cells. GOQD-HA-Met treatment for 6 h in PA induced RAW264.7 cells was able to downregulate the expression of these genes significantly showing anti-inflammatory efficacy in vitro. (Data represented as Mean ± SEM (n = 6) where *p < 0.05, **p < 0.01 and ***p < *0.001*. PA = palmitic acid, Met = Metformin, GH = GOQD-HA and GHM = GOQD-HA-Met.)

Next, to examine the beneficial effect of GOQD-HA-Met on inflammation induced by palmitic acid in RAW264.7 cells we performed quantitative RT-PCR to determine mRNA expression levels for proinflammatory cytokines/ chemokines (IL1b, TNFa, IL-6, MCP1, MIP-1a) (Fig. 4B–F), mediators of inflammation (NLRP3, INOS) (Fig. 4G, H), and immune cell marker (CD11c) (Fig. 4I). We observed that the expression of IL-1b and TNF-a was significantly reduced in the GOQD-HA-Met group compared to palmitate treated cells. Although the expression of IL-6 was reduced in the drug-loaded nanoconjugate group, there was no statistically significant difference compared to the palmitate group. The expression of NLRP3 and INOS also followed the same pattern where there was an increased expression of these two genes in the palmitate group which was subdued in the GHM group. The expression of the proinflammatory macrophage cell surface marker CD11c was also lowered significantly in the GHM group than the PA-treated group indicating the shift to a lesser inflammatory state in the cells treated with GHM.

We then evaluated the antioxidant efficacy of the metformin-loaded GOQD-HA in RAW264.7 cells by DCFDA staining (Fig. 4A). Upon incubating the cells with 250uM palmitic acid the amount of ROS generation was significantly elevated as observed by a higher intensity of the DCDFDA fluorescence intensity as compared to the untreated control group. Whereas the ROS generation was subdued significantly by treating the cells with GOQD-HA-Met when compared to the palmitic acid treated group.

### Metformin-loaded GOQD-HA mitigates adipose tissue inflammation and improves antioxidant status in DIO mice

**Fig. 5:**
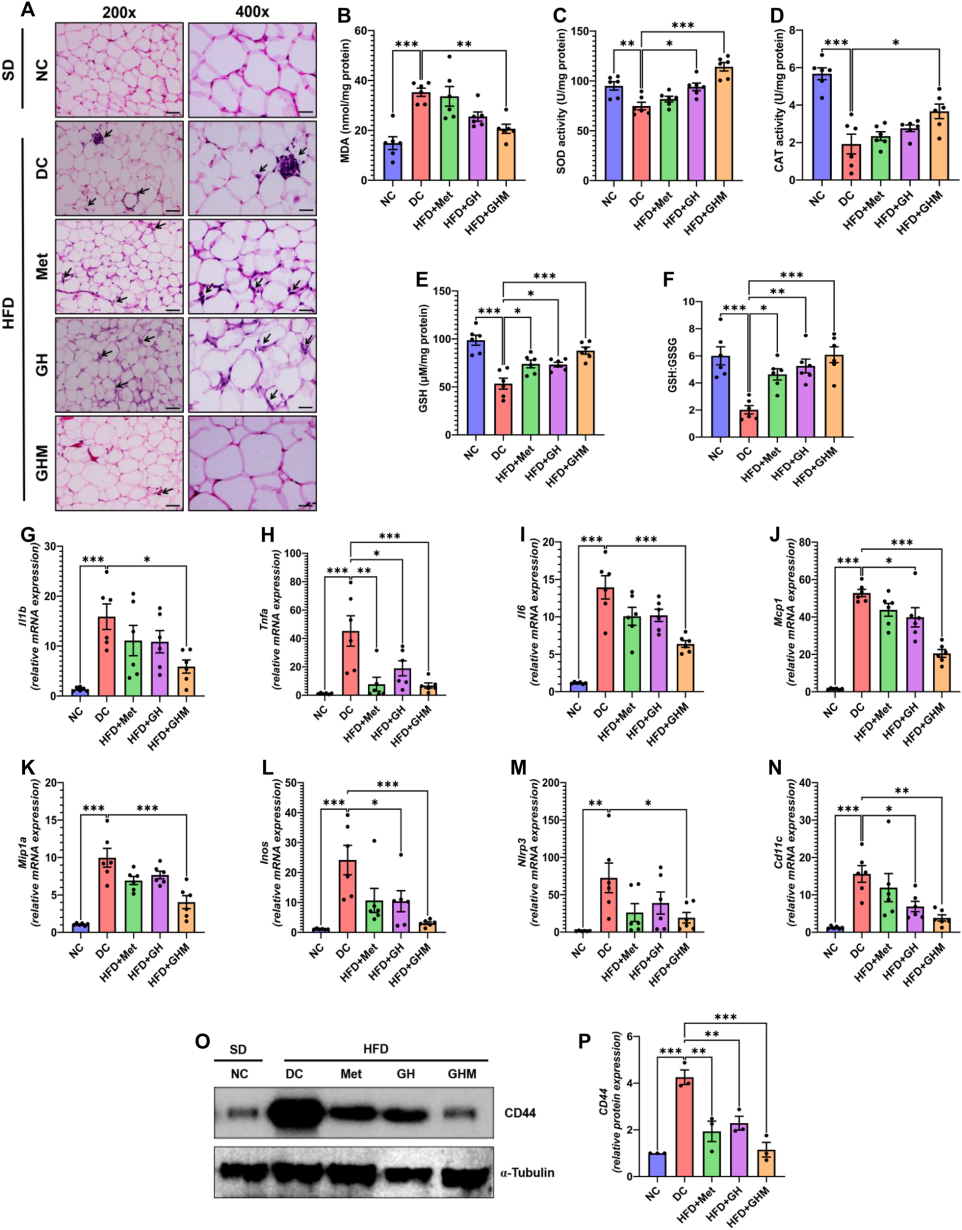
**GOQD-HA-Met efficiently attenuated HFD induced adipose tissue inflammation.** **A.** Histopathological study showing reduction in immune cell infiltration in eWAT of GOQD-HA-Met treated mice compared to eWAT of DC group of mice after 6 weeks of treatment (Black arrows indicating “crown like structures” or immune cell infiltration). **B.–F**. evaluation of enzymatic and nonenzymatic antioxidant markers from eWAT of different treatment groups after 6 weeks treatment. **B**. reduction in MDA levels and improvement of enzymatic activity of **C**. SOD, **D**. Catalase, **E**. GSH and **F**. GSH:GSSG ratio in GOQD-HA-Met group compared to DC group. **G.**–**K**. Effect of GOQD-HA-Met treatment on proinflammatory cytokine (**G**. Il1b; **H**. Tnfa; **I**. Il6) and chemokine (**J**. Mcp1; **K.** Mip1a) mRNA expression. **L.**–**N**. GOQD-HA-Met significantly reduced the expression of proinflammatory mediators (**L**. Inos; **M**. Nlrp3) and cell surface marker (**N**. Cd11c). **O**. Immunoblot showing the expression of CD44 in eWAT of different treatment groups. **P.** relative densitometry of CD44 protein expression from eWAT samples. (Data represented as Mean ± SEM (n = 3–6) where *p < 0.05, **p < 0.01 and ***p < *0.001*. NC = Normal Control, DC = Diabetic Control, HFD = High Fat Diet, Met = Metformin, GH = GOQD-HA and GHM = GOQD-HA-Met.)

We collected eWAT samples from all the mice and performed histopathological analysis (Fig. 5A). Immune cell infiltration in the form of an increased number of “crown-like structures” (CLSs) was present in the adipose tissue of HFD fed DC mice, while the number of CLS was markedly reduced in case of GOQD-HA-Met treated HFD fed mice. The number of CLSs was also lowered in both metformin and GH treated groups but the changes are not as significant as the GOQD-HA-Met group when compared to the DC group.

So, the results of the histopathological study led to the investigation of the underlying cause of the decreased immune cell infiltration in adipose tissue of the GOQD-HA-Met treated group. For this purpose, the mRNA expression of some key proinflammatory cytokines/ chemokines (IL1b, TNFa, IL-6, MCP1, MIP-1a), mediators of inflammation (NLRP3, INOS), and an immune cell marker (CD11c) in the adipose tissue of all the mice was measured by quantitative RT-PCR (Fig. 5G–N). It was observed that similar to in vitro experiments, here also eWAT of the DC mice had much higher expression of IL1b (Fig. 5G), TNFa (Fig. 5H), and IL-6 (Fig. 5I). The MCP-1 and MIP-1a (Fig. 5J, K) mRNA expressions were also elevated in the same group in line with the increased immune cell infiltration observed in the histopathological study of the eWAT. The expression levels of these cytokines and chemokines were decreased in all three groups (Metformin, GOQD-HA, and GOQD-HA-Met) but the changes in expression were more significant in the GOQD-HA-Met group when compared to the DC group than the other two treatment groups (Metformin and GOQD-HA). A similar pattern was observed for the expression of INOS (Fig. 5L) and NLRP3 (Fig. 5M) where the GOQD-HA-Met group had much lower expression than the NS group indicating the overall improvement of the inflammatory state of the eWAT upon treatment with GOQD-HA-Met in HFD fed mice. Interestingly, immunoblotting revealed that the expression of cell surface marker CD44 (Fig. 5O) is elevated significantly in the eWAT of the DC group. While the expression of CD44 was more significantly lowered in the GOQD-HA-Met group than Metformin and GOQD-HA groups (Fig. 5P). CD44 is mainly expressed on the cell surface of monocytes and macrophages. Increased expression of this cell surface receptor signifies increased immune cell infiltration in the adipose tissue in HFD fed DC group, all the while is subdued upon treatment with GOQD-HA-Met supporting the data found in histopathological studies and the gene expression study of proinflammatory macrophage marker Cd11c (Fig. 5N).

We further investigated oxidative stress markers (MDA) and antioxidant enzyme activity (SOD, Catalase, GSH) in all the mice from each treatment group. First, the MDA levels were significantly increased in the adipose tissue of the DC group compared to SD fed group (Fig. 5B). Interestingly the MDA levels decreased to within normal limits in the GOQD-HA-Met group, thus signifying lesser oxidative stress in the adipose tissue of this group of mice. Due to chronic exposure to HFD, increased oxidative stress for a longer period in the adipose tissue of the DC group resulted in impairment of the key antioxidant enzyme (SOD, catalase, and GSH) activity (Fig. 5C**–**F). Whereas, the activity of these enzymes was restored to their normal level after 6 weeks of treatment of the HFD fed mice with GOQD-HA-Met. The activity of SOD and GSH was also significantly elevated in GOQD-HA treated group too.

### HFD induced Non-Alcoholic Steatohepatitis (NASH) is alleviated by GOQD-HA-Met

**Fig. 6:**
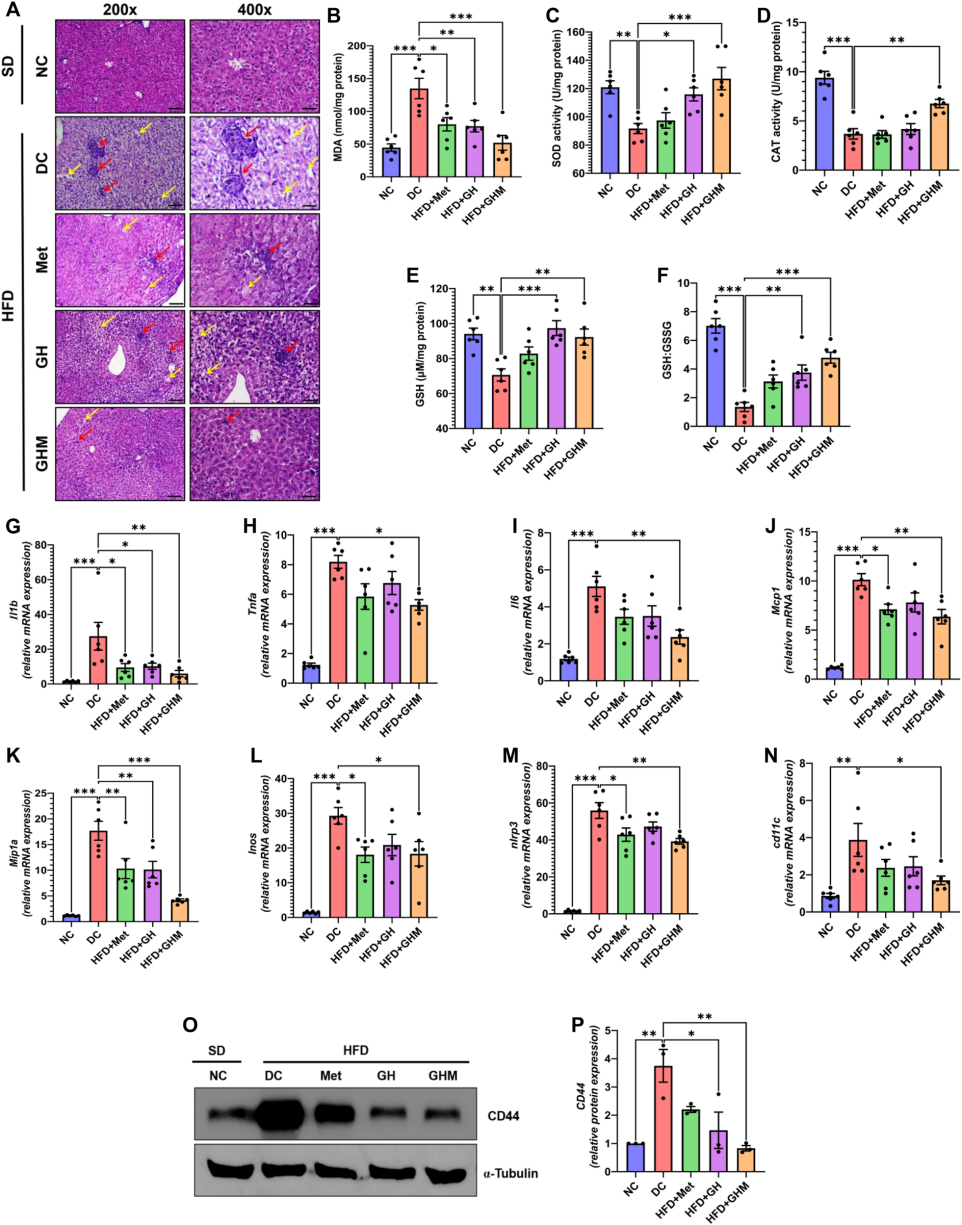
**HFD induced NASH related hepatic inflammation is alleviated by GOQD-HA-Met.** **A**. Histopathological study showing reduction in immune cell infiltration in liver of GOQD-HA-Met treated mice compared to liver biopsy sample of DC group of mice after 6 weeks of treatment (Red arrows indicating immune cell infiltration; Yellow arrows indicating ballooning degeneration). **B.**–**F**. Status of enzymatic and nonenzymatic antioxidant markers, evaluated from liver of different treatment groups after 6 weeks treatment. **B**. reduction in MDA levels and improvement of enzymatic activity of **C**. SOD, **D**. Catalase, **E**. GSH and **F**. GSH:GSSG ratio in GOQD-HA-Met group compared to DC group. **G.**–**K**. Effect of GOQD-HA-Met treatment on proinflammatory cytokine inliver samples (**G**. Il1b; **H**. Tnfa; **I**. Il6) and chemokine (**J.** Mcp1; **K.** Mip1a) mRNA expression. **L.**–**N**. GOQD-HA-Met significantly reduced the expression of proinflammatory mediators (**L.** Inos; **M.** Nlrp3) and cell surface marker (**N**. Cd11c). **O**. Immunoblot showing the expression of CD44 in eWAT of different treatment groups. **P**. relative densitometry of CD44 protein expression from eWAT samples. (Data represented as Mean ± SEM (n = 3–6) where *p < 0.05, **p < 0.01 and ***p < *0.001*. NC = Normal Control, DC = Diabetic Control, HFD = High Fat Diet, Met = Metformin, GH = GOQD-HA and GHM = GOQD-HA-Met)

Histopathological investigation of the liver samples from all the mice was performed to assess the therapeutic effect of the nanoconjugate in HFD induced hepatic injury (Fig. 6A). From this study it was evident that there were degenerative changes in the liver of HFD fed mice. Histopathology of liver biopsy samples from DC mice showed characteristics of steatohepatitis: ballooning degeneration, focal immune cell infiltration, and fibrosis. Whereas, the treatment with Metformin and GOQD-HA-Met both reversed the deleterious effects of HFD more significantly than only GOQD-HA treated mice. Although focal immune cell aggregation was reduced significantly in all three treatment groups when compared to NS treated group.

In line with these findings the mRNA expression of proinflammatory cytokines (IL-1b, TNF-a, and IL-6) was significantly upregulated in the liver of HFD fed NS treated mice. We found that GOQD-HA-Met treatment was able to bring down the expression of IL-1b (Fig. 6G**.**), TNF-a (Fig. 6H), and IL-6 (Fig. 6I) significantly. A similar pattern was observed in the mRNA expression of Mcp1 and Mip1a (Fig. 6J**–**K). On the other hand, the heightened expression of INOS and NLRP3 in the liver of DC mice was mitigated in both Metformin and GOQD-HA-Met-treated mice significantly. We also carried out quantitative RT-PCR for proinflammatory macrophage cell surface marker CD11c (Fig. 6N) in the mice of each treatment group but there were no significant changes found in the expression level of this cell surface marker in the GOQD-HA and metformin group but, GOQD-HA-Met was able to decrease the expression of this cell surface marker. Although the immunoblotting of cell surface marker CD44 (Fig. 6O) showed a significant decrease in protein expression in both GOQD-HA and GOQD-HA-Met groups, but not in the Metformin group (Fig. 6P).

To gain further insight into the therapeutic efficacy of the metformin-loaded GOQD-HA nanocomposite first we evaluated oxidative stress marker (MDA) and antioxidant enzyme (SOD, catalase, GSH) activity status in the liver tissue of all the mice of each treatment group (Fig. 6B**–**F). Similar to the result found in eWAT, liver tissues in the HFD fed DC mice recorded a significant elevation in MDA levels when compared to the SD fed control group (Fig. 6B). The MDA level was lowered significantly in all three treatment groups. As a consequence, of the high MDA level, the activity of the antioxidant enzymes (SOD, catalase, and GSH) was impaired in liver samples of the DC group. While the activity of SOD (Fig. 6C) and GSH (Fig. 6E) was restored to normalcy in both GOQD-HA and GOQD-HA-Met treated groups when compared to DC mice.

Thus, the antioxidant and anti-inflammatory action of the drug-loaded nanocomposite GOQD-HA-Met resolved the lipid induced liver injury or steatosis. This phenomenon became more apparent when we performed liver function tests on plasma samples from all the mice. There was a heightened activity of liver enzymes (ALT, ALP and AST) (Additional file 1: Fig. S3A–C) in the plasma of the DC group indicating damage to hepatic tissue. Whereas, these parameters came down within the normal range more significantly in the GOQD-HA-Met group when compared to the DC group than the other two treatment groups (metformin and GOQD-HA).

### GOQD-HA-Met improves systemic chronic low-grade inflammation in HFD fed mice

We next tried to explore whether our synthesized nanoconjugate GOQD-HA-Met has any effect on the HFD induced systemic inflammation by performing ELISA of the proinflammatory cytokines (IL-1b, IL-6 and TNF-a) (Additional file 1: S4A–C) from plasma samples of all the mice. We found that the DC group had elevated proinflammatory cytokine levels in the plasma that were mitigated by treatment with GOQD-HA-Met. The metformin-treated group was also able to reduce the levels of IL-1b and IL-6 but not the TNF-a in the plasma. We also performed indirect ELISA for the detection of CD44 (Additional file 1: Fig. S4D) from the plasma of all the mice in the different treatment groups. The result showed a similar pattern as the proinflammatory cytokines except for the fact that here GOQD-HA treatment was better at lowering the level of CD44 than the Metformin group when compared with the DC group.

### Improvement of adipose tissue inflammation and NASH after GOQD-HA-Met treatment leads to the betterment of glycemic status and other metabolic parameters in HFD fed mice

**Fig. 7:**
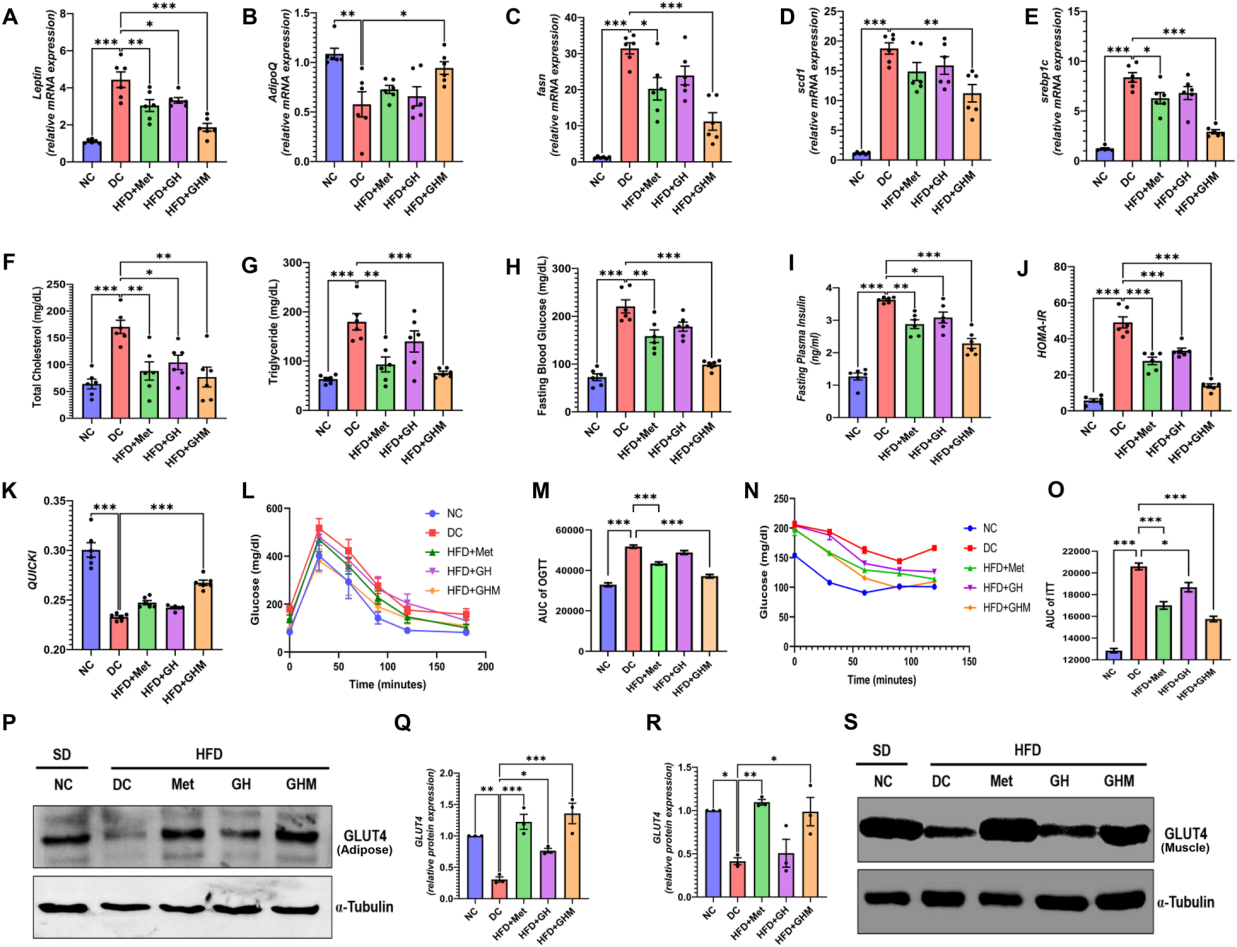
**Beneficial effect of GOQD-HA-Met on systemic inflammation and metabolic parameters in HFD induced diabetic mice.** **A**. Leptin & **B**. Adipoq mRNA expression profile improvement in GOQD-HA-Met treated mice. **C.**–**E**. Effect of GOQD-HA-Met on mRNA expression of de novo lipid synthesis pathway genes in liver; **C**. Fasn, **D**. Scd1, **E**. Srebp1c. **F**. Serum Total Cholesterol and **G**. Serum triglyceride levels in mice were reduced in GOQD-HA-Met treatment group. **H**. Fasting blood glucose and **I**. Fasting plasma Insulin levels in different groups of mice. **J.** HOMA-IR and **K.** QUICKI scores improved in GOQD-HA-Met group compared to DC group. **L.**–**O**. GOQD-HA-Met treatment lead to betterment of insulin resistance as represented by **L.** OGTT, **M** AUC of OGTT, **N.** ITT, **O**. AUC of ITT in mice. **P.**–**S**. Evaluation of GLUT4 protein expression for confirmation of reversal of insulin resistance in GOQD-HA-Met treated mice. **P.** Representative immunoblot of GLUT4 from eWAT. **Q.** Quantification of GLUT4 protein expression from eWAT. **R.** Representative immunoblot of GLUT4 from muscle. **S.** Quantification of GLUT4 protein expression from muscle. (Data represented as Mean ± SEM (n = 3–6) where *p < 0.05, **p < 0.01 and ***p < *0.001*. NC = Normal Control, DC = Diabetic Control, HFD = High Fat Diet, Met = Metformin, GH = GOQD-HA and GHM = GOQD-HA-Met)

After evaluating the anti-inflammatory potential of metformin-loaded graphene oxide quantum dots hyaluronic acid nanoconjugate, we tried to discern how this reversal of chronic low-grade inflammation in adipose and liver affected the different metabolic parameters in HFD fed diabetic mice. First of all, we evaluated Leptin expression levels in adipose tissue by quantitative RT-PCR (Fig. 7A**.**). Where we observed that changes in Leptin mRNA levels were decreased in all three treatment groups but the degree of the changes was more statistically significant in the GHM group. While adiponectin expression (Fig. 7B) was significantly increased only in the GOQD-HA-Met group compared to the DC group. On the other hand, increased fat intake and subsequent insulin resistance led to increased expression of genes involved in de novo lipid synthesis (Fasn, Scd1, and Srebp1c) in the liver of HFD fed DC mice (Fig. 7C–E). Interestingly, as the inflammation was resolved, the expression of these genes was reduced in all the treatment groups compared to the DC group, and the changes were statistically significant only in metformin and GOQD-HA-Met treated mice for Fasn and Srebp1c and only in GOQD-HA-Met for Scd1. As a result, improvement in the serum TC (Fig. 7F) and TG (Fig. 7G) levels were noticed in both Metformin and GOQD-HA-Met treated groups than in the DC group.

Fasting blood glucose level was lowered significantly in the metformin and GOQD-HA-Met groups compared to the DC group after 6 weeks of treatment (Fig. 7H). FBG was reduced in the GOQD-HA group too, but the changes were not statistically significant. Moreover, serum insulin (Fig. 7I) and HOMA-IR (Fig. 7J) score were also improved significantly in metformin GOQD-HA and GOQD-HA-Met groups. But QUICKI scores improvement was statistically significant only in GOQD-HA-Met treated mice when compared to DC mice (Fig. 7K). In line with these results, we performed OGTT and ITT in all the mice after 6 weeks of the treatment protocol. Here we observed that the metformin and GOQD-HA-Met group showed better oral glucose tolerance in the OGTT experiment (Fig. 7L, M). On the other hand, all the treatment groups showed significant betterment of insulin-mediated glucose homeostasis in ITT experiments (Fig. 7N, O). Finally, the reversal of insulin resistance was confirmed via protein expression analysis by immunoblot of GLUT-4 in eWAT (Fig. 7P, Q) and muscle (Fig. 7R, S). It was observed that in the case of DC mice, the expression of GLUT4 was diminished significantly compared to the SD fed NC group in both eWAT and muscle implying the development of insulin resistance. In adipose, the GLUT4 expression was restored to normal levels in Metformin and GOQD-HA-Met more significantly than GOQD-HA. Whereas, in muscle GLUT4 expression was restored to the level as high as the SD fed NC group only in Metformin and GOQD-HA-Met treated mice signifying overall systemic improvement of insulin sensitivity.

## Discussion

Adipose tissue inflammation and NASH in the obese conditions are often linked with the advent of insulin resistance where the proinflammatory cytokines and chemokines directly or indirectly exert a negative influence on the insulin signaling pathway. The adipose tissue macrophages (ATM) orchestrate the events of the shifting to the inflammatory milieu in the first place, as a result of oxidative stress, mechanical stress, and hypoxic conditions in response to the dysfunctional WAT expansion due to obesity [56]. Polarization of the ATM into a proinflammatory condition further produces chemokines that attract circulatory monocytes and other immune cells into adipose tissue which further worsens the inflammation and leads to chronic systemic low-grade inflammation or meta-inflammation. This meta-inflammation coupled with lipotoxicity induced by adipose tissue fat spillage contributes to the progression of hepatic steatosis. In this case, the liver also starts producing inflammatory cytokines that exacerbate the overall inflammatory state in the body along with hepatic tissue injury characterized by fibrosis and degeneration [57–59]. To make things worse this meta inflammatory state along with AT inflammation and hepatic steatosis leads the way to insulin resistance and eventual progression to T2DM.

**Fig. 8:**
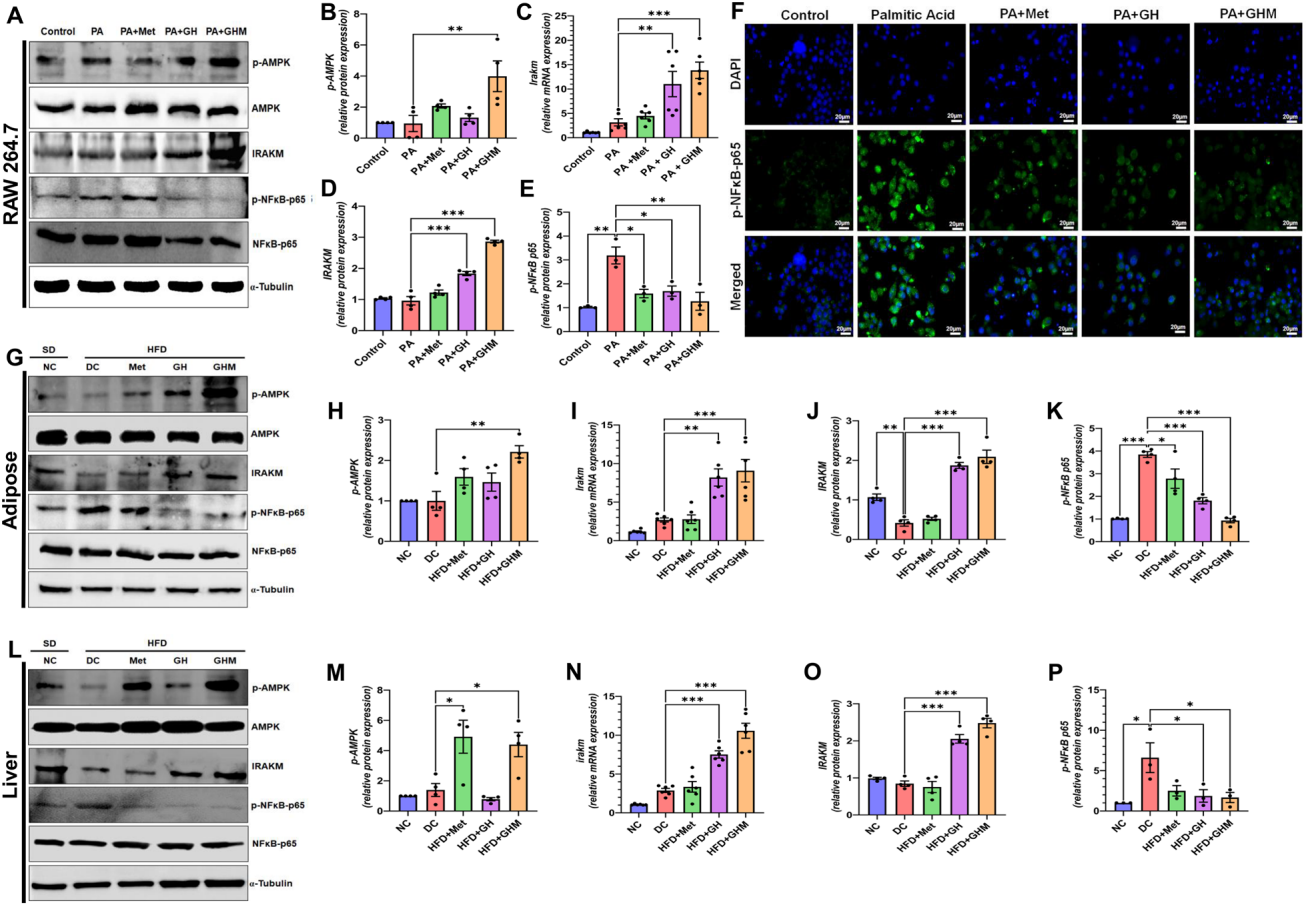
**GOQD-HA-Met exerts its anti-inflammatory efficacy via dual action of IRAKM and AMPKα.** **A.** Representative immunoblots of phospho-AMPK, AMPK, IRAKM, phospho-NF-κB-p65, NF-κB-p65 and α-Tubulin from RAW264.7 cell lysates. **B**–**E**. quantification of protein and mRNA expression from RAW264.7 cell lysates. **B**. Phosphorylated AMPKα protein expression. **C** Irakm mRNA expression, **D** IRAKM protein expression, **E**. Phosphorylated NF-κB p65 protein expression. **F** Immunocytochemistry for phospho-NF-κB-p65 showing increased phosphorylation of p65 in PA induced RAW264.7 cells that is subsequently subdued in Metformin, GOQD-HA and GOQD-HA-Met groups. **G**. Representative immunoblots of phospho-AMPK, AMPK, IRAKM, phospho-NF-κB-p65, NF-κB-p65 and α-Tubulin from eWAT homogenates. **H** –**K**. quantification of protein and mRNA expression from eWAT. **H**. Phosphorylated AMPKα protein expression. **I** Irakm mRNA expression, **J.** IRAKM protein expression, **K** Phosphorylated NF-κB p65 protein expression. **L** Representative immunoblots of phospho-AMPK, AMPK, IRAKM, phospho-NF-κB-p65, NF-κB-p65 and α-Tubulin from liver homogenates. **M**–**P**. quantification of protein and mRNA expression from liver. **M**. Phosphorylated AMPKα protein expression. **N** Irakm mRNA expression, **O**. IRAKM protein expression, **P**. Phosphorylated NF-κB p65 protein expression. (Data represented as Mean ± SEM (n = 3–6) where *p < 0.05, **p < 0.01 and ***p < *0.001*. PA = Palmitic Acid, NC = Normal Control, DC = Diabetic Control, HFD = High Fat Diet, Met = Metformin, GH = GOQD-HA and GHM = GOQD-HA-Met)

As such, our aim was to evaluate the anti-inflammatory efficacy of blank GOQD-HA and GOQD-HA-Met compared to free metformin. In our study mice were fed HFD to induce adipose tissue inflammation, NASH and IR in murine models. Similarly, PA was used for in vitro inflammation model development. PA is the most abundant free fatty acid found in the plasma of obese patients and it is also a TLR4 against making it a potent activator of inflammatory pathways. Here, our HFD induced DIO mice model demonstrated the induction of pro-inflammatory cytokines (IL1b, TNF-a, IL6, Mcp1) in adipose tissue and the liver. Induction of inflammatory cytokines also activates NLRP3 inflammasome due to ectopic fat deposition [60]. PA induced cellular inflammatory model showed impairment of the same signaling pathway. We found that the GOQD-HA-Met efficiently mitigated the PA induced inflammation in RAW264.7 cells and HFD induced inflammation in adipose tissue and the liver as well. Similarly, metformin and blank GOQD-HA treated groups also showed a marked decrease in the mRNA levels of the inflammatory genes studied but this decrease was not as significant as that of the drug-loaded nanoconjugate. Previous studies have reported that the upregulation of proinflammatory cytokines and chemokines in macrophages induced by PA stress is mainly mediated via NF-κB [61, 62]. Similarly, NF-κB activation is also implicated in the progression of adipose tissue inflammation and NASH in both murine and human studies. Under normal conditions, NF-κB is a heterodimeric protein complex, sequestered in the cytoplasm in an inactive form by IκB. However, NF-κB is activated upon stimulation by different extracellular (proinflammatory cytokines, TLR activation) and intracellular signals (ER stress, ROS generation, DNA damage) via IκK-mediated phosphorylation of IκB and its subsequent proteasomal degradation. Now the dimeric NF-κB (p65 and p50) complex is also phosphorylated at Ser536 of the p65 subunit [63, 64] and then translocates to the nucleus to transactivate the transcription of numerous genes related to the inflammatory pathway [65, 66]. In our study, we have observed a significant increase in p65 phosphorylation in both PA treated RAW264.7 cells (Fig. 8A) and in the AT and liver of HFD fed DC mice (Fig. 8G, L), thus indicating NF-κB activation. The p65 phosphorylation was almost nullified in GOQD-HA-Met treated RAW264.7 cells (Fig. 8E), as well as in the AT (Fig. 8K) and liver (Fig. 8P) of the HFD fed mice, implying the resolution of the inflammatory state in both in vitro and in vivo studies. For further validation we evaluated the mRNA expression of Iκb in RAW264.7 cells; also, in mice adipose tissue and liver samples (Additional file 1: Fig. S5A–C). Iκb transcription is regulated by NF-κB as a feedback loop where more NF-κB activation leads to more expression of IκB which subsides when the external or internal stimulus of IκK activation are subdued. Similar pattern was found in our study where there was a significant increase in Iκb mRNA expression in PA treated cells and, in the tissue, (adipose and liver) of HFD fed mice compared to control. Whereas, the GOQD-HA-Met treated group showed more significant decrease in the IκB expression than metformin and GOQD-HA treated groups, a pattern similar to the phospho p65 expression. This study thus adds weight to the evidence that the GOQD-HA-Met treatment resolves the proinflammatory signaling via inactivation of the NF-κB signaling pathway. We then further tried to understand the underlying mechanism by which GOQD-HA-Met downregulates NF-κB activation.

**Fig. 9:**
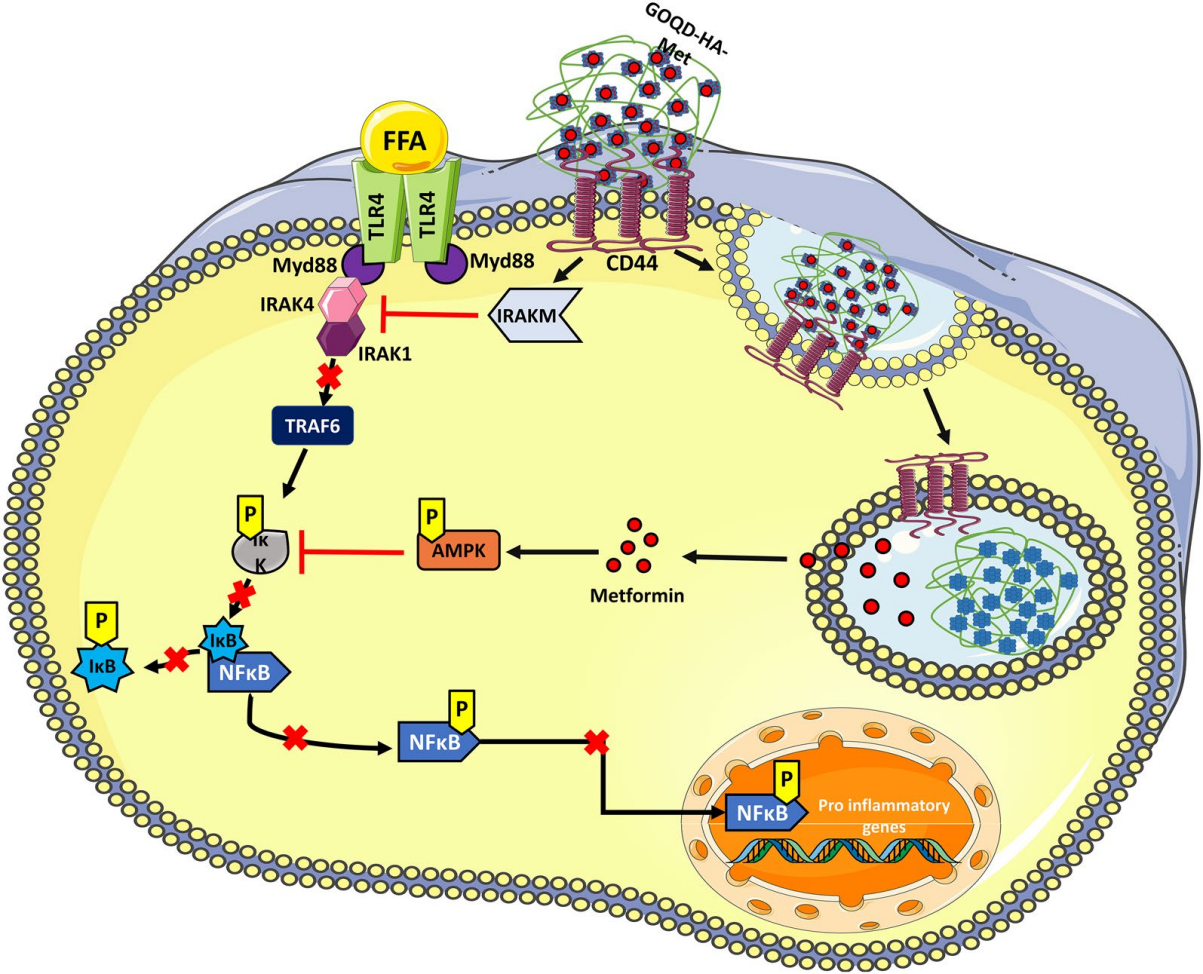
**Proposed anti-inflammatory mechanism of action of GOQD-HA-Met on free fatty acid induced activated macrophages.** Free fatty acid levels increase in case of obesity induced adipose tissue inflammation and NASH. This FFA is a potent perpetrator involved in activating macrophages via TLR4 into proinflammatory state. FFA upon binding with TLR4 activates IRAK4 in downstream signalling via Myd88. Activated IRAK4 then further activates IRAK1 which in turn phosphorylates TRAF6 and thereby activating IκK. IκK phosphorylation leads to inhibitory phosphorylation of IκB thereby dissociating it from NF-κB and thus activating NF-κB. IκK also phosphorylates p65 (one of the components of NF-κB dimeric complex) upon which NF-κB complex enters nucleus and upregulates various genes involved in proinflammatory response (cytokines, chemokines). Now GOQD-HA-Met actively targets the proinflammatory macrophages via CD44 through hyaluronic acid. This CD44 and GOQD-HA-Met interaction via hyaluronic acid activates IRAKM which prevents dissociation of IRAK1 from IRAK4, so that IRAK1 cannot activate TRAF6, and thus indirectly blocks the activation of IκK. On the other hand, the GOQD-HA-Met is internalized by receptor mediated endocytosis and metformin is released intracellularly. Now Metformin activates AMPKα which also prevents NF-κB activation by inhibition of IκK activation. Our synthesized nanocomposite inhibits NF-κB

AMPK plays a key role in metabolic energy regulation at the cellular level [67] and metformin is reported to downregulate NF-ΚB activation via activation of AMPK [68]. Therefore, we hypothesized that our metformin-loaded quantum dot formulation could have a role through the mediation of AMPK regulation. First, we performed immunoblotting for phospho-AMPK in both RAW 264.7 cells and mice tissues (Liver and Adipose) from all the treatment groups (Fig. 8A, G and L). We observed that AMPK was significantly activated by phosphorylation only in the metformin and GOQD-HA-Met groups but not in the GOQD-HA group both in vitro and in vivo conditions (Fig. 8B, H and M). On the other hand, HMW-HA has also been reported to downregulate NF-κB activation thus showing anti-inflammatory effects. HA upon binding with CD44 induces the expression of IRAK-M, which acts as a negative regulator of TLR/IL1R mediated pro-inflammatory pathway [69]. In our study IRAK-M mRNA (Fig. 8C) and protein expression (Fig. 8D) was significantly upregulated in blank GOQD-HA and GOQD-HA-Met groups but not in the metformin treated group in RAW264.7 cells. A similar expression pattern was recorded in AT (Fig. 8I and J) and Liver (Fig. 8N and O) of HFD fed mice. This implies that the treatment groups containing hyaluronic acid in its composition were only able to induce the expression of IRAK-M in both in vitro and in vivo conditions. PA is the most abundant free fatty acid in the case of obesity and is a TLR4 agonist. After ligand binding of TLR/IL1R, the Myd88/IRAK4 complex is formed and IRAK4 phosphorylates IRAK1. IRAK-M binds with IRAK4 thus preventing phosphorylation-mediated activation of IRAK1 and hence hindering TRAF6/IRAK1 complex formation. TRAF6/IRAK1 complex initiates IΚK-mediated activation of NF-ΚB. Thus, blocking the TRAF6/IRAK1 complex formation via inhibition of IRAK4 by IRAK-M indirectly downregulates the NF-κB activation [70, 71]. So, from these experiments it is evident that GOQD-HA-Met treated groups have both upregulated AMPK phosphorylation and IRAK-M expression suggesting a synergistic action of both metformin and HA that resulted in the most efficient downregulation of NF-κB in PA treated RAW264.7 cells and DIO mice (Fig. 9). In that context, our nano-formulation also explores an alternative route of metformin delivery in target tissue via CD44 using HA as a targeting agent through the dual-mode action where HA upon binding with CD44 downregulates NF-κB through the IRAK-M pathway, subsequently upon releasing of metformin it also downregulates NF-κB via AMPK activation.

On the other hand, GOQD-HA-Met also improved antioxidant status through the inhibition of ROS generation and restoring antioxidant enzyme activity. It is a well-known fact that inflammation and oxidative stress go hand in hand, and if one is suppressed other one is subdued as well. So, the efficient mitigation of inflammation by GOQD-HA-Met under in vitro and in vivo conditions might have played a significant role in rescuing the cells and tissues from oxidative damage. However, it is worth mentioning here that in recent studies GOQDs have been shown to have antioxidant properties [72, 73] and there are some reports of HA being an antioxidant agent as well [74]. Therefore, our synthesized nanoconjugate with both GOQDs and HA in its composition may have contributed to the improvement of oxidative stress along with amelioration of inflammation in the GOQD-HA-Met group and as well as in the blank GOQD-HA group.

These findings suggest that the GOQD-HA nanocarrier itself has therapeutic potential that enhances the efficacy of metformin in suppressing the inflammatory response in PA and HFD-induced stress in RAW264.7 cells and diabetic mice respectively. Finally, the resolution of inflammation and restoration of oxidative homeostasis resulted in the betterment of glycemic and metabolic status in DIO mice, thereby improving insulin sensitivity as evident through the upregulation of GLUT4 expression in both adipose tissue and muscle of GOQD-HA-Met treated DIO mice.

Numerous earlier researches have examined various nano formulations for metformin delivery in type 2 diabetes [75–77]. Since metformin exhibits poor bioavailability and necessitates greater doses, the majority of studies solely employ the nanoformulation as a carrier with a primary focus on the sustained release of the medication [78–80]. In our investigation, loading metformin onto the GOQD-HA increased the drug's targetability and bioavailability, which was demonstrated by the fact that lower dosages were needed to achieve the same effects as free metformin. Additionally, we have proven that the nanocarrier GOQD-HA acts as a therapeutic agent itself, boosting the overall effectiveness of metformin in case of meta-inflammation and associated diabetic complications. In light of this, we can say that our metformin-loaded graphene oxide quantum dots hyaluronic acid nanoconjugate has a competitive advantage over other nanoformulations of metformin that focus solely on the sustained release aspect of drug delivery.

However, GOQD-HA, a nanocarrier, demonstrates the therapeutic effectiveness of itself in the case of metainflammation. The key factor is the presence of GOQD, which has antioxidant characteristics [73], and HMW hyaluronic acid, which has anti-inflammatory properties. In addition, because GOQDs are graphene based materials and have more side groups than GQDs, they can be easily functionalized with other macromolecules [81]. While the same planar graphene structure and side groups that include OH and COOH groups make it a better option for drug loading [82]. The majority of studies using a graphene-based nanomaterial conjugated with hyaluronic acid are used in the field of chemotherapeutic drug delivery because hyaluronic acid can be used to target the CD44 receptor, which is frequently overexpressed in cases of cancer [39, 83–85]. But in this instance, we have repurposed the nanoconjugate in the event of metainflammation. Due to its inherent therapeutic characteristics, hyaluronic acid nanoparticles are now being researched in a variety of inflammatory diseases, including arthritis, acute kidney damage, and atherosclerosis [23, 25, 86–89]. However, no drug molecules were incorporated in the NP in those investigations; instead, only HA NP was employed as a therapeutic agent. However, in our situation, the presence of GOQD allows for the loading of various pharmacological or antioxidant molecules into the nanocomposite, which may then be used as a dual-purpose CD44 targeted drug delivery platform in which the nanoconjugate itself serves as a therapeutic agent.

## Conclusion

We have synthesized a CD44-targeted Hyaluronic acid conjugated Graphene oxide quantum dots (GOQD-HA) nanocomposite loaded with antidiabetic drug metformin (Met) for the treatment of adipose tissue inflammation, NASH, and IR. GOQD-HA-Met efficiently alleviated high fat diet induced adipose tissue inflammation and NASH thereby reversing insulin resistance. The drug loaded nanocomposite primarily worked through downregulation of NF-κB activation by dual mode pincer action of IRAKM and AMPK via HA and Metformin respectively. Our experimental evidences established that the GOQD-HA nanocomposite as a drug delivery system that has a therapeutic potential on its own and can also serve as a targeted carrier for the drug metformin to enhance its efficacy for the treatment of obesity-induced meta-inflammation of adipose tissue, and liver thereby improving the treatment outcome of patients with T2DM. Therefore, GOQD-HA coupled with metformin or other types of anti-inflammatory or antioxidant drugs can be studied as a CD44-mediated therapeutic strategy in cases of other inflammatory diseases as well.

## Supplementary Information

**Additional file 1: Table S1**: List of primers used for quantitative RT-PCR. **Figure S1**: **A.** UV-Vis spectrum of GO; **B.** UV-Vis spectrum of GOQD; **C.** Photoluminescence spectra of GOQD; **D. – F.** Zeta potential of GOQD, GOQD-HA and GOQD-HA-Met. **Figure S2**: Metformin release profile from GOQD-HA-Met at pH 2, 5.4, 7.4 and 12. **Figure S3**: Liver function test parameters: **A. **Serum ALT; **B. **Serum ALP; **C. **Serum AST. **Figure S4**: Serum inflammatory marker levels after 6 weeks treatment in mice; **A. **IL1β; **B. **TNFα; **C. **IL6; **D. **CD44. **Figure S5**: Relative mRNA expression of Iκb in **A.** RAW264.7; **B.** Adipose; **C.** Liver.

## Data Availability

The raw/processed data required to reproduce these findings will be made available on request.
