# Supplementary material for: Hyaluronic acid-graphene oxide quantum dots nanoconjugate as dual purpose drug delivery and therapeutic agent in meta-inflammation
Source: J Nanobiotechnology. 2023 Aug 1;21:246. doi: 10.1186/s12951-023-02015-w (PMC10394801; doi:10.1186/s12951-023-02015-w)
Supplement: Supplementary file 1 — Additional file 1: Table S1: List of primers used for quantitative RT-PCR. Figure S1: A. UV-Vis spectrum of GO; B. UV-Vis spectrum of GOQD; C. Photoluminescence spectra of GOQD; D. – F. Zeta potential of GOQD, GOQD-HA and GOQD-HA-Met. Figure S2: Metformin release profile from GOQD-HA-Met at pH 2, 5.4, 7.4 and 12. Figure S3: Liver function test parameters: A. Serum ALT; B. Serum ALP; C. Serum AST. Figure S4: Serum inflammatory marker levels after 6 weeks treatment in mice; A. IL1β; B. TNFα; C. IL6; D. CD44. Figure S5: Relative mRNA expression of Iκb in A. RAW264.7; B. Adipose; C. Liver. [file 12951_2023_2015_MOESM1_ESM.pdf]

Additional file

Hyaluronic Acid-Graphene Oxide Quantum Dots nanoconjugate as dual purpose drug delivery and therapeutic agent in meta-inflammation

Kunal Sarkar[1], Sarbashri Bank[1], Arindam Chatterjee[1], Koushik Dutta[2], Anwesha Das[1], Santanu Chakraborty[1], Nirvika Paul[1], Jit Sarkar[5], Sriparna De[3], Sudakshina Ghosh[4], Krishnendu Acharyya[5], Dipankar Chattopadhyay[2], Madhusudan Das[1]*

[1]Department of Zoology, University of Calcutta, 35 Ballygunge Circular Road, Kolkata- 700019, India.

[2]Department of Polymer Science and Technology, University of Calcutta, 92 A.P.C. Road, Kolkata- 700009, India.

[3]Department of Allied Health Sciences, Brainware University, Kolkata-700129, India.

[4]Department of Zoology, Vidyasagar College for Women, Kolkata-700006, India.

[5]Molecular and Applied Mycology and Plant Pathology Laboratory, Department of Botany, University of Calcutta, Kolkata -700019, India

***Correspondence:** madhuzoo@yahoo.com

Additional file

Additional file 1: Table 1: List of primers used for quantitative RT-PCR

Gene	**Forward Primer**	**Reverse Primer**
Il1b	GGTGTGTGACGTTCCCATTA	ATTGAGGTGGAGAGCTTTCAG
Tnfa	CTGAGTTCTGCAAAGGGAGAG	CCTCAGGGAAGAATCTGGAAAG
Il6	CTTCCATCCAGTTGCCTTCT	CTCCGACTTGTGAAGTGGTATAG
Mcp1	CTCACCTGCTGCTACTCATTC	ACTACAGCTTCTTTGGGACAC
Mip1a	TCACTGACCTGGAACTGAATG	CAGCTTATAGGAGATGGAGCTATG
Nlrp3	CTTCCATGGCTCAGGACATAC	CAACGGACACTCGTCATCTT
Inos	GTGGCTGTGCTCCATAGTT	CCGGTACTCATTCTGCATGT
Cd11c	AGCAGCCATGACCAGTTTAC	TCTCTCTGGCCCAGGTTATT
Irakm	GAATAGCCAAAGCCATCCAATAC	GAGCTGGTCATCCAAGAGTATG
Leptin	GCACCCTATGTCACCATCAA	CAGAGCTGAGCACGAAACT
Adipoq	ACTTGTGCAGGTTGGATGG	TCTCCTTTCTCTCCCTTCTCTC
Fasn	CAACCGGCTCTCTTTCTTCT	CCTGGTAGGCATTCTGTAGTG
Scd1	TAGCACCTTCTTGCGATACAC	CTCCCGGGATTGAATGTTCTT
Srebp1c	AAGCGCTACCGGTCTTCTAT	TTTATTCAGCTTTGCTTCAGTGC
Ikb	CCTTCCTCAACTTCCAGAACAA	GATCACAGCCAGCTTTCAGA
Gapdh	AACAGCAACTCCCACTCTTC	CCTGTTGCTGTAGCCGTATT

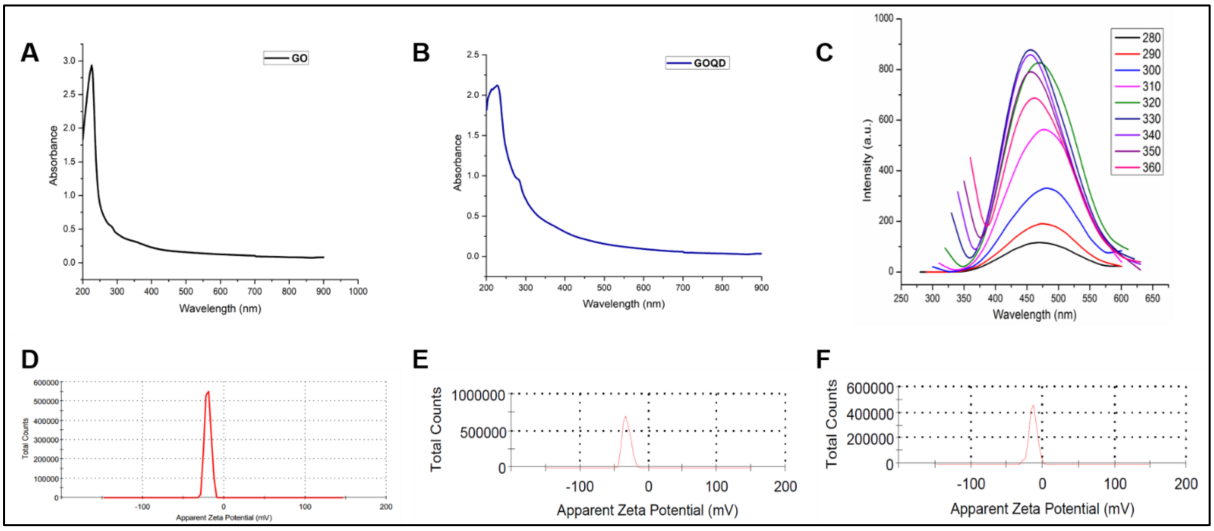

Additional file 1: Figure S1: A. UV-Vis spectrum of GO; B. UV-Vis spectrum of GOQD; C. Photoluminescence spectra of GOQD; D. – F. Zeta potential of GOQD, GOQD-HA and GOQD-HA-Met

Additional file

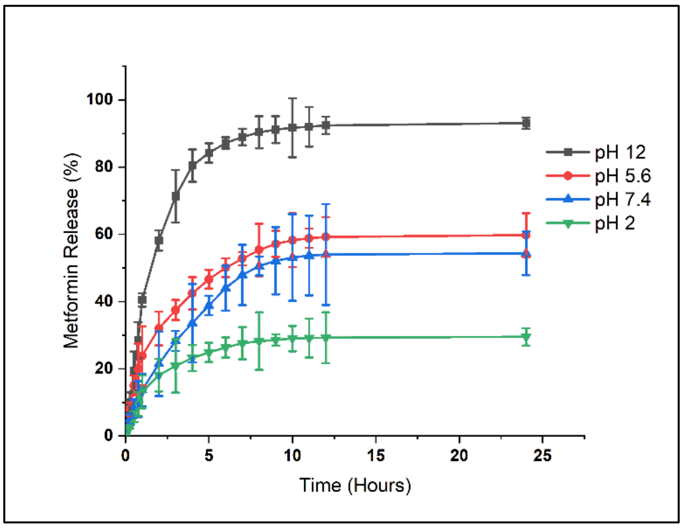

Additional file 1: Figure S2: Metformin release profile from GOQD-HA-Met at pH 2, 5.4, 7.4 and 12.

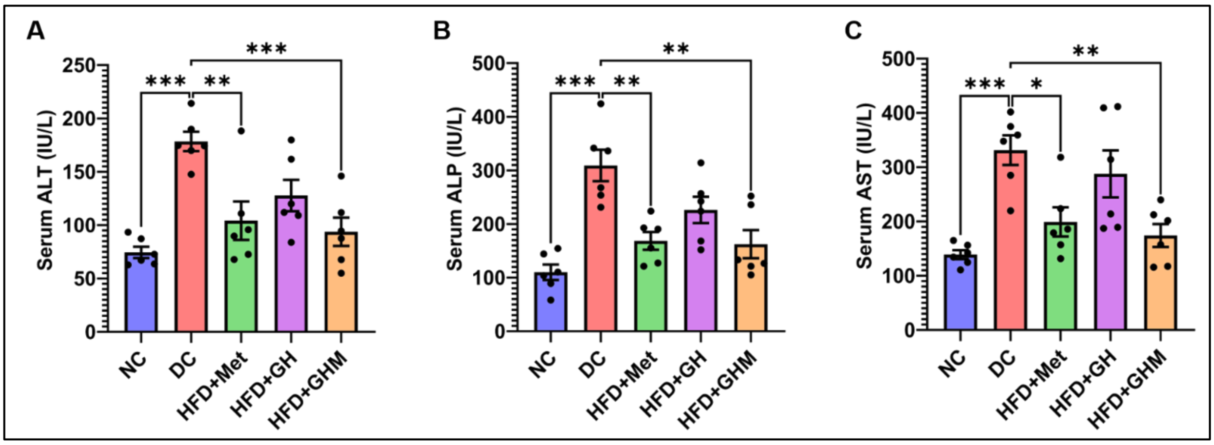

Additional file 1: Figure S3: Liver function test parameters: **A.** Serum ALT; **B.** Serum ALP; **C.** Serum AST

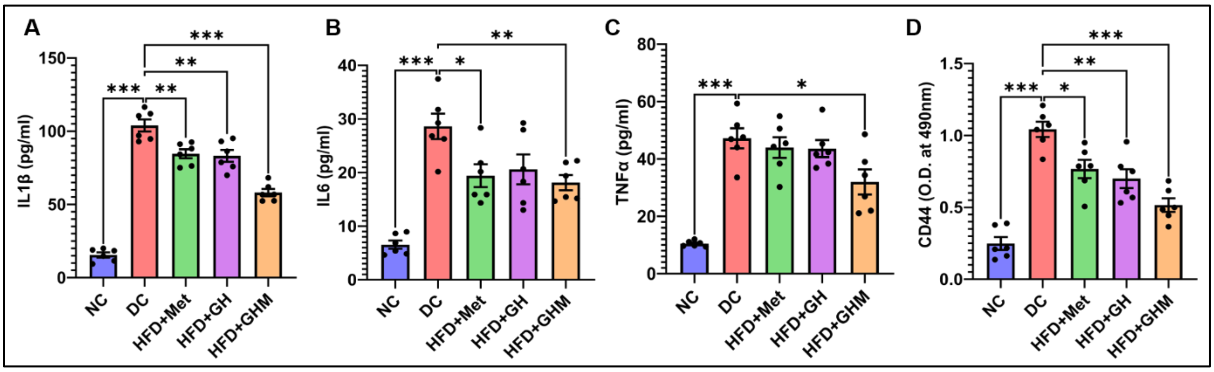

Additional file 1: Figure S4: Serum inflammatory marker levels after 6 weeks treatment in mice; **A.** IL1β; **B.** TNFα; **C.** IL6; **D.** CD44

Additional file

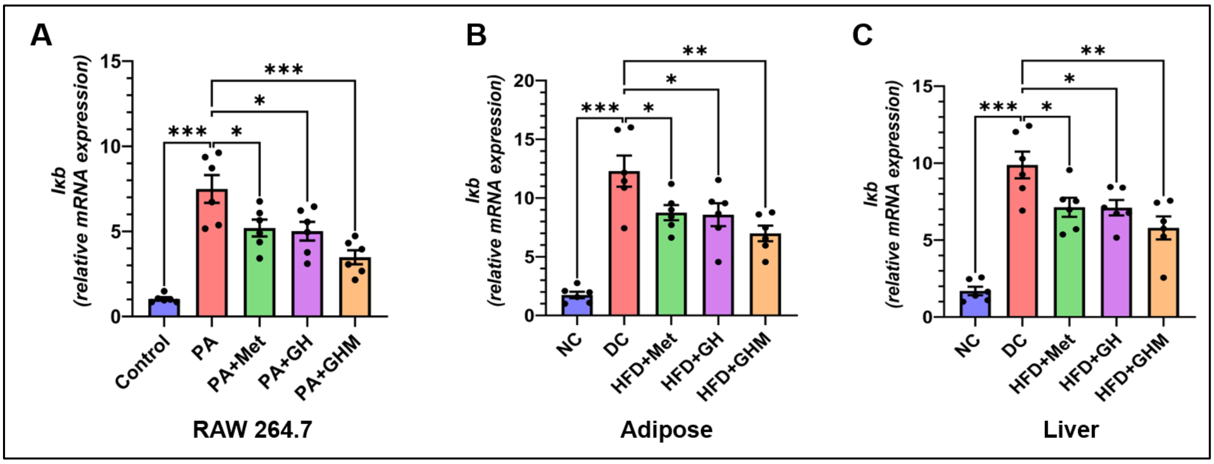

Additional file 1: Figure S5: Relative mRNA expression of Iκb in A. RAW264.7; B. Adipose; C. Liver
